# Integrated bioinformatics analysis to decipher molecular mechanism of compound Kushen injection for esophageal cancer by combining WGCNA with network pharmacology

**DOI:** 10.1038/s41598-020-69708-2

**Published:** 2020-07-29

**Authors:** Wei Zhou, Jiarui Wu, Jingyuan Zhang, Xinkui Liu, Siyu Guo, ShanShan Jia, Xiaomeng Zhang, Yingli Zhu, Miaomiao Wang

**Affiliations:** Beijing University of Chinese Medicine, Beijing, 100102 China

**Keywords:** Bioinformatics, Biochemical networks

## Abstract

Compound Kushen injection (CKI), a medicine in widespread clinical use in China, has proven therapeutic effects on cancer. However, few molecular mechanism analyses have been carried out. To address this problem, bioinformatics approaches combining weighted gene co-expression network analysis with network pharmacology methods were undertaken to elucidate the underlying molecular mechanisms of CKI in the treatment of esophageal cancer (ESCA). First, the key gene modules related to the clinical traits of ESCA were analysed by WCGNA. Based on the results, the hub genes related to CKI treatment for ESCA were explored through network pharmacology. Molecular docking simulation was performed to recognize the binding activity of hub genes with CKI compounds. The results showed that the potential hub targets, including EGFR, ErbB2, CCND1 and IGF1R, are therapeutic targets of CKI for the treatment of ESCA. Moreover, these targets were significantly enriched in many pathways related to cancer and signalling pathways, such as the PI3K-Akt signalling pathway and ErbB signalling pathway. In conclusion, this research partially highlighted the molecular mechanism of CKI in the treatment of ESCA, offering great potential in the identification of the effective compounds in CKI and biomarkers for ESCA treatment.

## Introduction

Esophageal cancer (ESCA) is widespread worldwide. According to Global Cancer Statistics 2018, it ranks seventh in incidence and sixth in mortality^1^. The 5-year survival rate of ESCA is between 12 and 20% and differs substantially by sex^2^. China is a high-risk area for ESCA, especially in some rural areas, where the incidence rate far exceeds that of urban areas due to lifestyle and environmental reasons^3^. ESCA can be divided into esophageal adenocarcinoma (EAC) and esophageal squamous cell carcinoma (ESCC) according to histological classification. In recent decades, the incidence of EAC in Western countries has increased several times, and the proportion of ESCC has exceeded 90% throughout China^4,5^. The introduction of chemo(radio)therapy and surgical therapy led to increased survival rates and reduced the incidence of recurrence^6^. Because conventional methods do not adequately improve patient survival of ESCA, however, scientists are seeking more effective treatments. Recently, traditional Chinese medicine (TCM) has taken the world stage as complementary and alternative medicine^7^. Compound Kushen Injection (CKI) consists of two herbs, Kushen (Radix Sophorae Flavescentis) and Baituling (Rhizoma Smilacis Glabrae). CKI mainly contains various anticancer ingredients, such as matrine and oxymatrine, which can inhibit the growth of tumour cells, overcome resistance to metastasis and multidrug resistance, and protect human immunity^8^. CKI has been utilized in clinical practice for decades to treat various solid tumour types, including liver cancer, breast cancer, gastric cancer, and other cancer types^8,9^. The analysis of medical data on 2,550 ESCA patients from 22 large-scale hospitals in China confirmed that CKI has been relatively widely used in the clinical treatment of ESCA of different severities^10^. In addition, a previous study reported that CKI limited cancer pain both directly by blocking TRPV1 signalling and indirectly by reducing tumour growth^11^. For ESCA, it is worth noting that CKI used alone or combined with conventional radiotherapy can not only enhance antitumor efficacy but also reduce the toxicity induced by radiotherapy, thereby improving the quality of life^12^. Our early studies also found that combination with CKI can improve the clinical effectiveness rate and performance status of radiotherapy for ESCA. Furthermore, CKI can also provide treatment by reducing gastrointestinal reactions and radiation esophagitis^13.^

Tumorigenesis is a complex process that is driven by a combination of networks of genes and environmental factors; there is a lack of effective methods to identify functional networks that Chinese medicine interferes with tumorigenesis^14–17^. To better analyse and predict the molecular mechanism of CKI in the treatment of ESCA, this study adopted weighted gene co-expression network analysis (WGCNA) integrated with the network pharmacology method. WGCNA can be used to find clusters (modules) of highly related genes, correlate modules and correlate with external sample traits and can be used to identify candidate biomarkers or therapeutic targets^18^. Network pharmacology not only caters to the “multi-component, multi-target” characteristics of TCM but also identifies drug-gene-disease links, explaining the therapeutic mechanism of drugs at the molecular level^19–21^.

**Figure 1:**
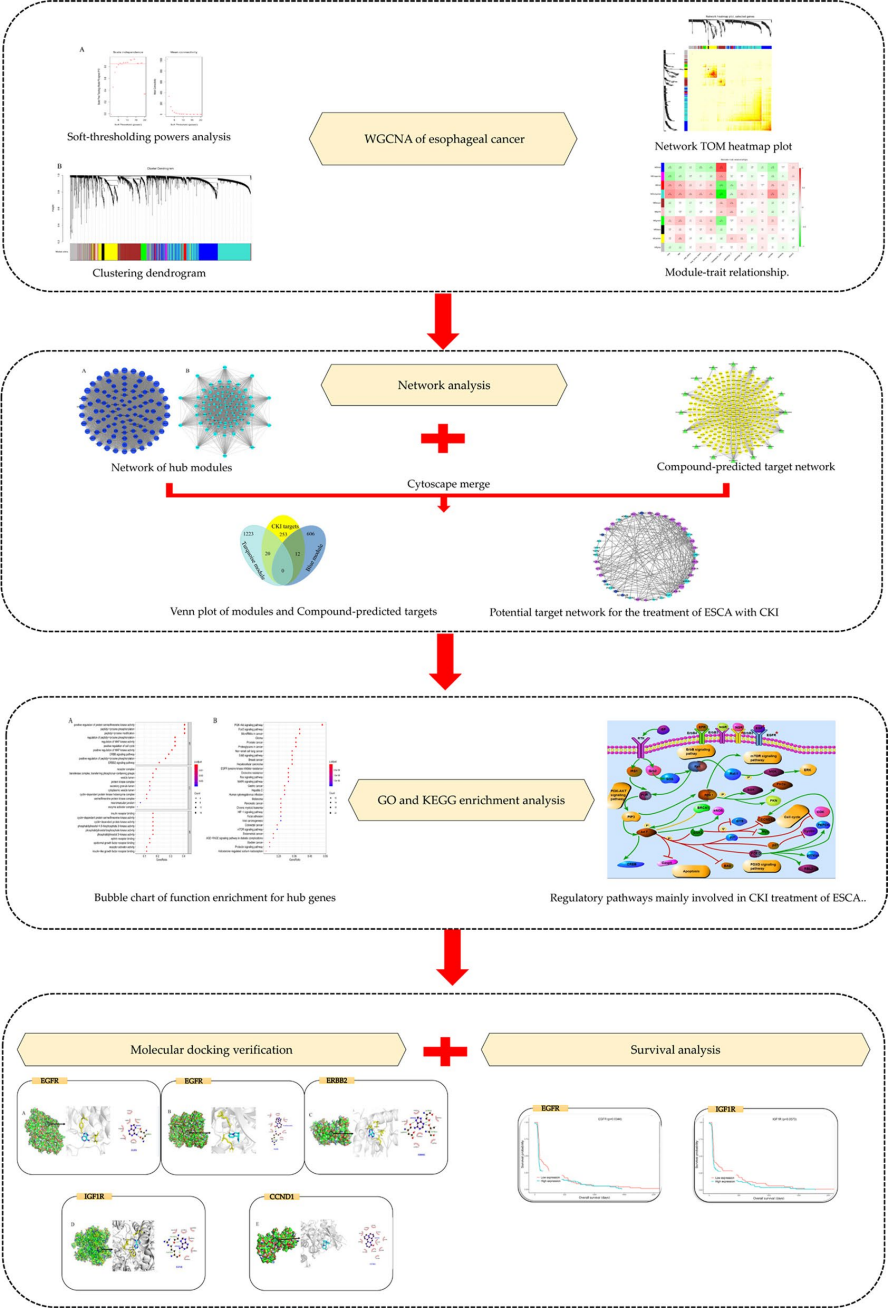
Workflow of this integrated bioinformatics analysis.

In the present work, we first used WGCNA to analyse ESCA mRNA datasets from The Cancer Genome Atlas (TCGA) to predict significant gene modules. Second, gene modules were combined with predicted targets of key CKI components to form a drug-gene-disease network and further analysed. In addition, molecular docking methods were adopted to confirm the degree of binding between the hub gene and the component. This study is intended to explain the mechanism of action of CKI in the treatment of ESCA at the molecular level and to provide a better basis for the diagnosis, treatment and prognosis of ESCA. Figure 1 depicts a flowchart of the technical strategy used in this study.

## Results

### WGCNA module construction

**Figure 2:**
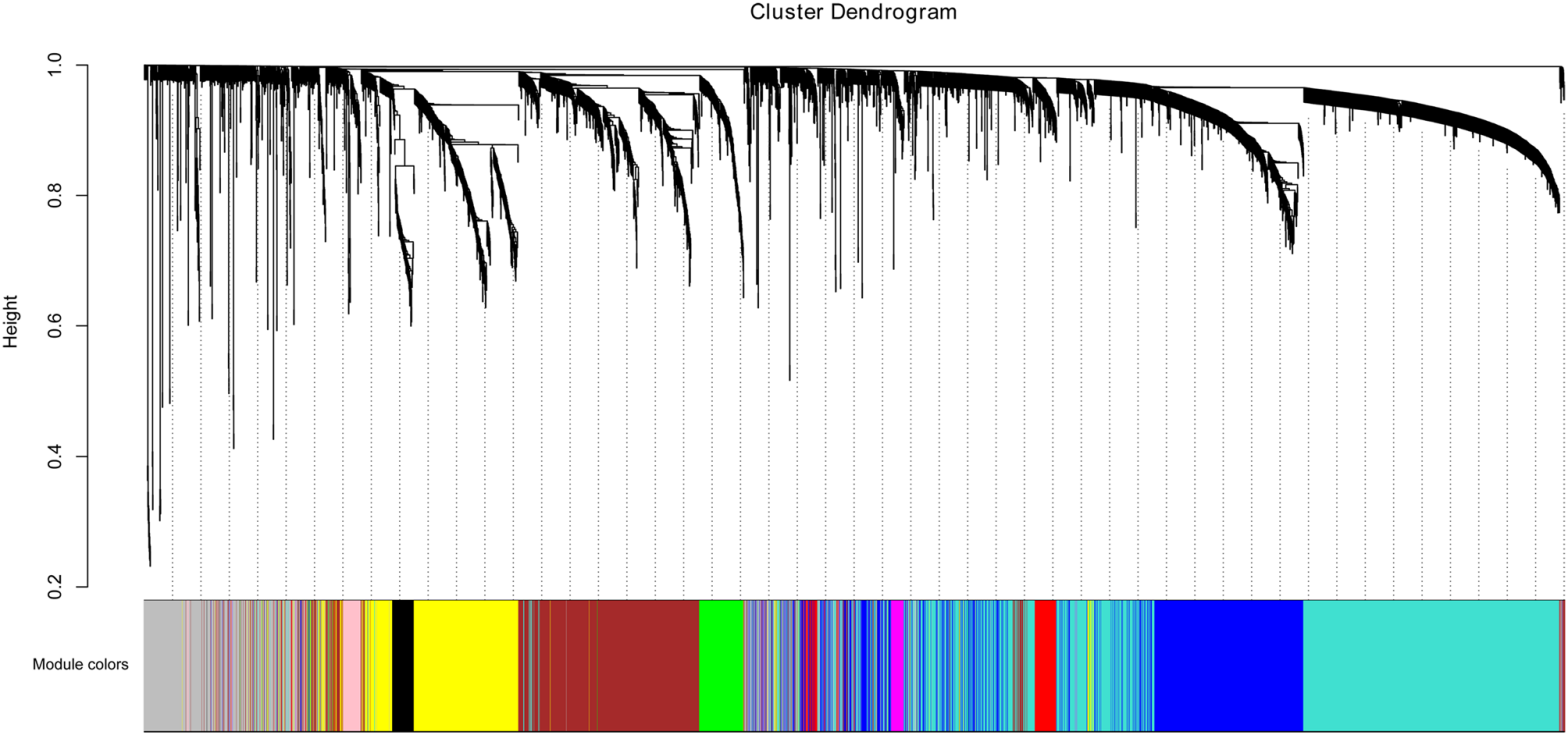
Clustering dendrogram. corFnc = “pearson”; power = 6; min. module size = 30; mergeCutHeight of 0.2.

A total of 161 samples and 5,000 genes were screened for the next WGCNA analysis. After normalization, no outlier samples were eliminated. In this study, the power of β = 6 (scale free *R*^2^ = 0.85) was selected as the soft-thresholding parameter to ensure a scale-free network. (Fig. 2A) A total of 10 modules were identified via average linkage hierarchical clustering (Fig. 2B).

### WGCNA hub module screening

**Figure 3:**
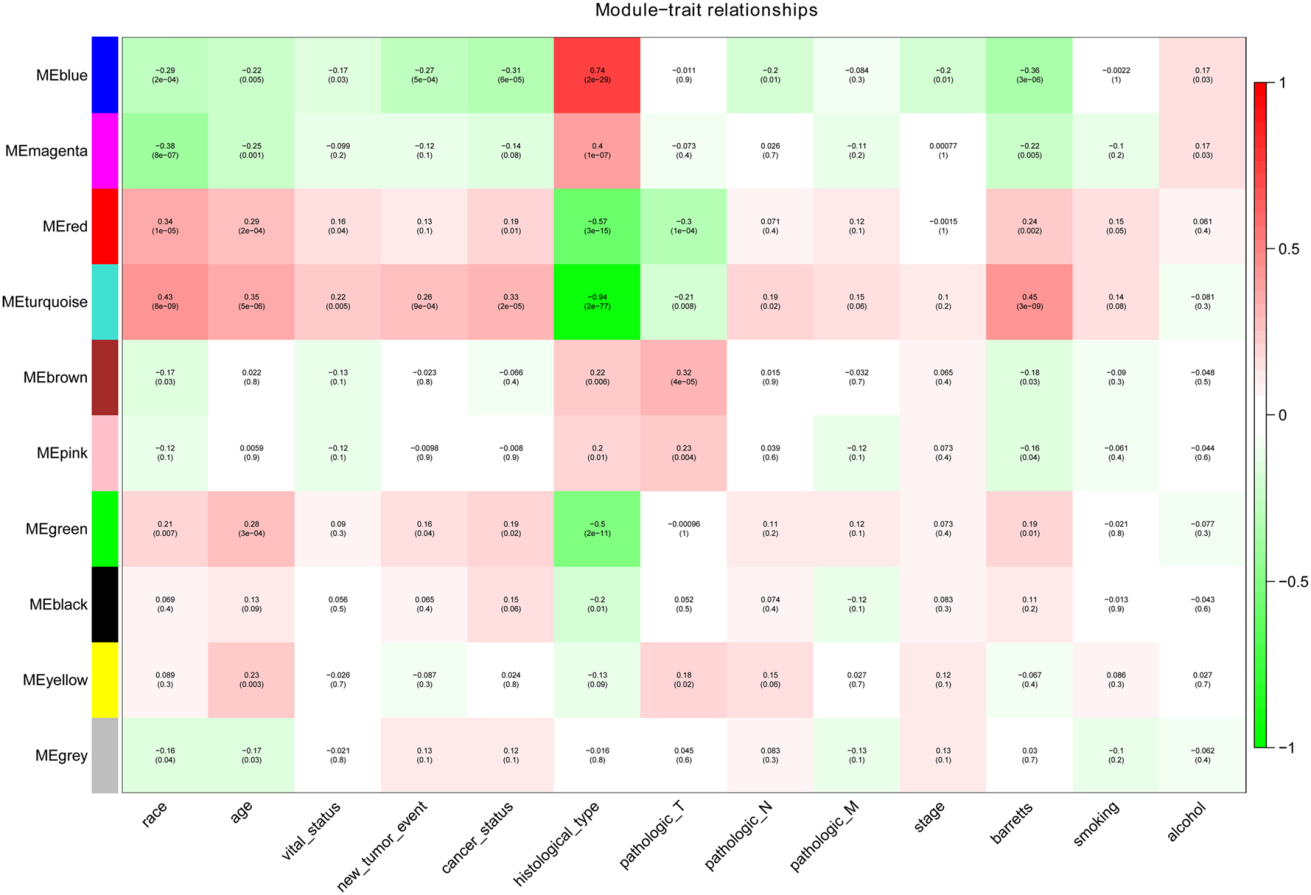
Module-trait relationship. Each row corresponds to an ME, and each column corresponds to a clinical trait. Each cell contains a corresponding correlation.

**Figure 4:**
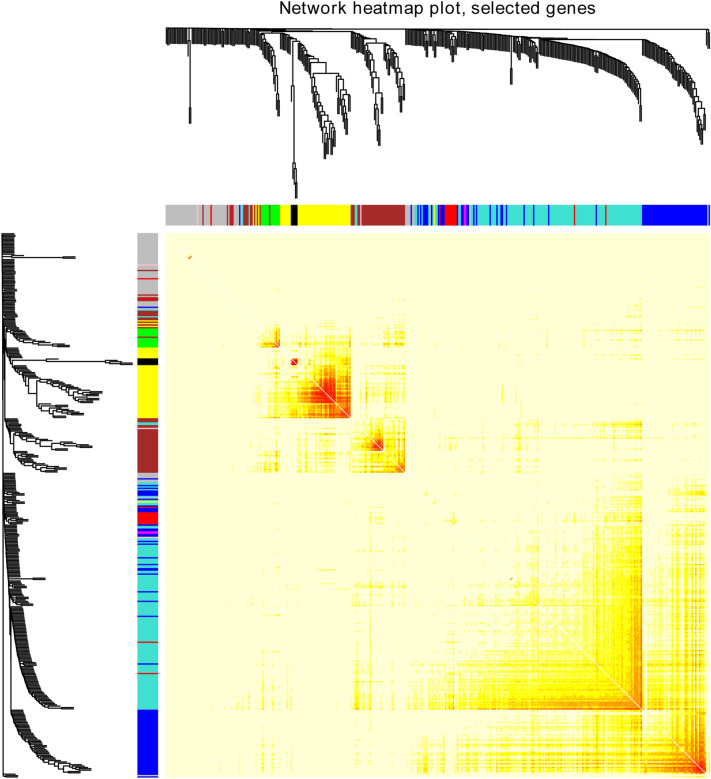
Network TOM heatmap plot. The TOM plot is made up of 400 randomly selected genes. Each row and column represents a module and the genes of the module. This diagram shows the degree of correlation within the module.

**Figure 5:**
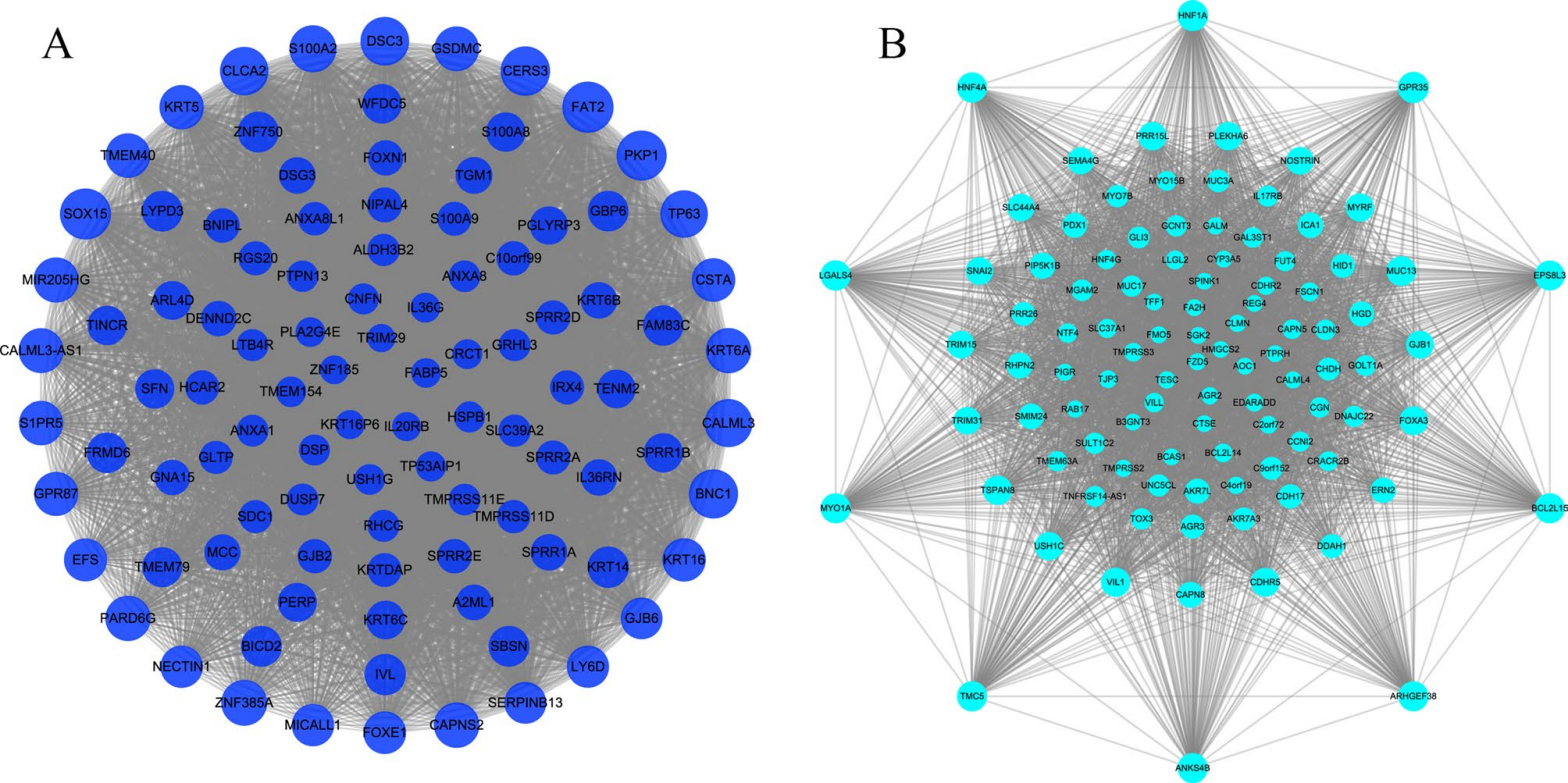
The top 100 genes in the degree of hub modules. (**A**) Blue module. (**B**) Turquoise module.

ME reflected the gene expression level of the entire module, and the relationship between ME and clinical traits was assessed by Pearson’s test. The module and clinical traits were considered statistically significant when *p* < *0.05.* The blue module and the turquoise module were considered to be hub modules through the association of modules with clinical traits (race, age, vital status, new tumour events, cancer status, histological type, pathologic T, pathologic N, pathologic M, stage, Barretts, smoking, alcohol) (Fig. 3). The topological overlap measure (TOM) was visualized with a heatmap that could depict adjacencies or topological overlaps (Fig. 4). Each module contained a set of RNAs that were co-expressed and had a high TOM. The same module genes could form networks and may participate in similar biological processes. The network building the key modules was filtered with a weight Cutoff = 0.1 between the genes. The blue module consists of 618 genes and 31,042 gene linkages. The turquoise module consists of 1,243 genes and 49,230 gene linkages. In addition, the top 100 genes in terms of degree were visualized using Cytoscape (Fig. 5).

### Compound-predicted target network

Following a literature search^8,22^, the 23 active ingredients contained in CKI were selected for investigation, and the three-dimensional structure data of 16 active ingredients were obtained from the PubChem database^23^.

**Figure 6:**
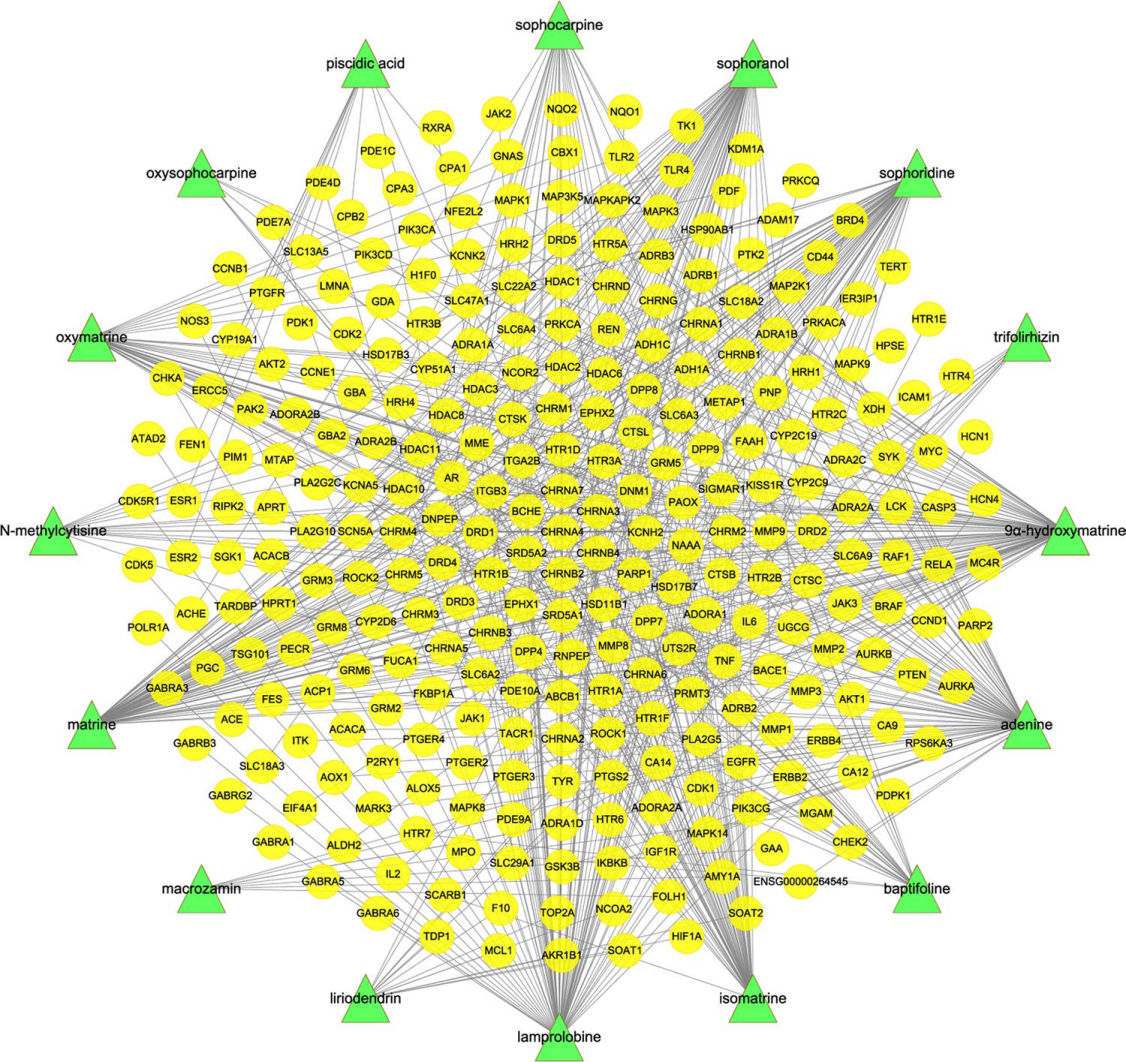
Compound-predicted target network. Sixteen compound nodes are green, and 285 target nodes are yellow.

The 16 active compounds of CKI are shown in Table 1. After Cytoscape visualization, 301 points (16 compound points and 285 gene points) and 636 edges were obtained (Fig. 6).

**Table 1:** Information on the active ingredients of CKI.

PubChem CID	Compound	Structure	PubChem CID	Compound	Structure
15,385,684	9α-Hydroxymatrine		87,752	Lamprolobine	
190	Adenine		226,371	Liriodendrin	
621,307	Baptifoline		9,576,780	Macrozamin	
5,271,984	Isomatrine		91,466	Matrine	
115,269	Sophocarpine		670,971	N-methylcytisine	
12,442,899	Sophoranol		24,864,132	Oxymatrine	
165,549	Sophoridine		24,721,085	Oxysophocarpine	
442,827	Trifolirhizin		6,710,641	Piscidic acid

### Potential target network for the treatment of ESCA with CKI

**Figure 7:**
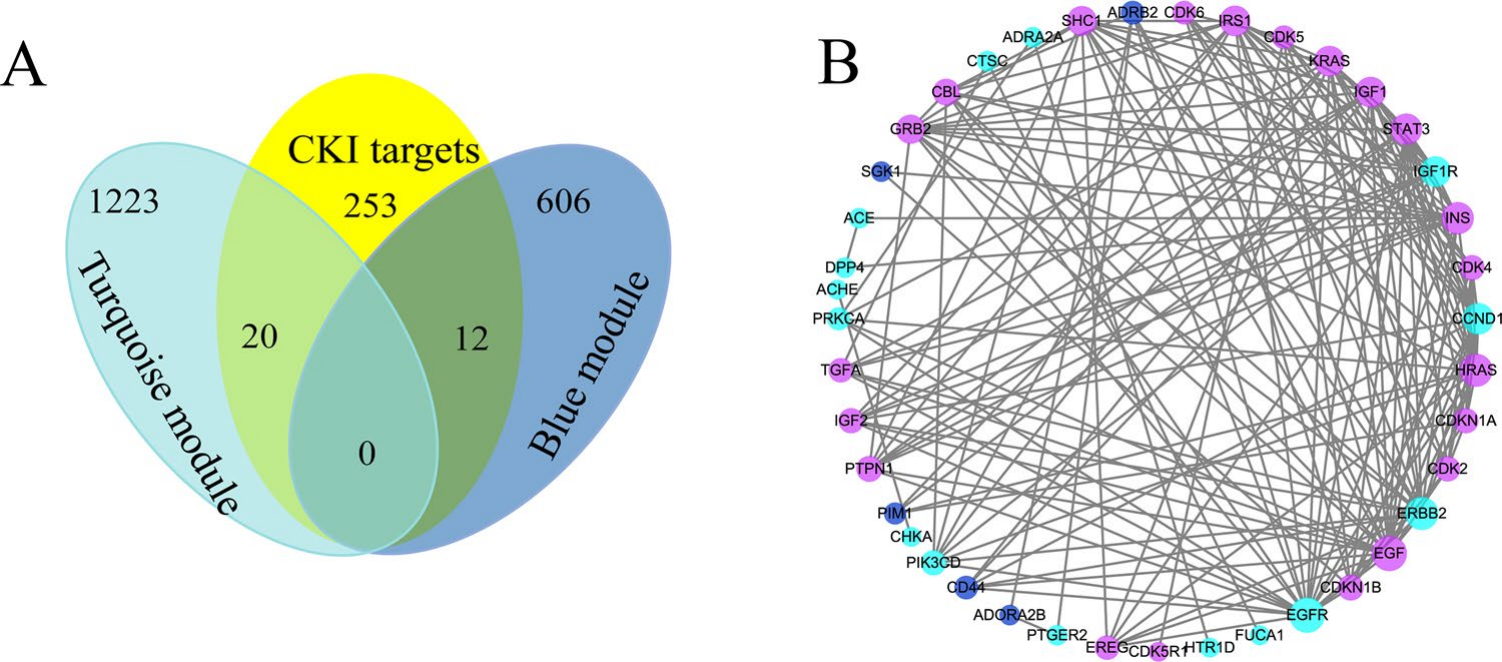
(**A**) Venn plot of modules and compound-predicted targets. (**B**) Potential target network for the treatment of ESCA with CKI. Blue and turquoise nodes indicate their corresponding colour module genes, and purple nodes represent PPI-related human targets.

The compound-predicted target network was combined with the blue and turquoise module genes. Thirty-two identical gene targets were considered potential targets for the CKI treatment of ESCA (Fig. 7A). To further unveil the therapeutic mechanism, STRING was used to construct a PPI network of 32 overlapping genes between the compound targets and key module targets. As shown in Fig. 7B, the potential therapeutic PPI network involved 41 nodes and 174 linkages between genes. Moreover, after network analysis, a target with a greater degree value than the mean based on the topological characteristics is a key gene for the CKI treatment of ESCA. The results of network analysis show 16 nodes with an average degree ≥ 8.49, including EGF, EGFR, ErbB2, HRAS, INS, STAT3, CCND1, IRS1, KRAS, IGF1R, IGF1, SHC1, GRB2, CBL, PTPN1 and CDKN1B.

### GO functional and KEGG pathway enrichment analysis

**Figure 8:**
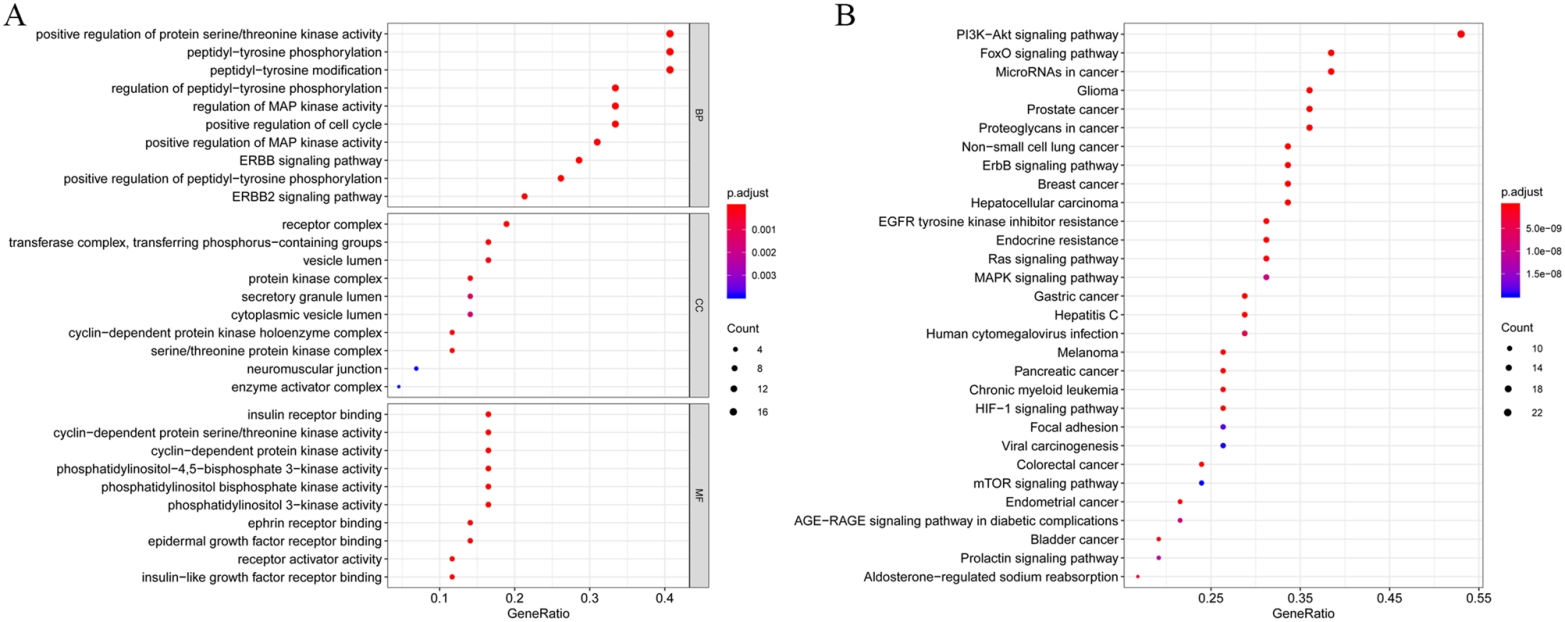
Bubble chart of functional enrichment for hub genes. (**A**) GO function enrichment (**B**) KEGG function enrichment.

**Figure 9:**
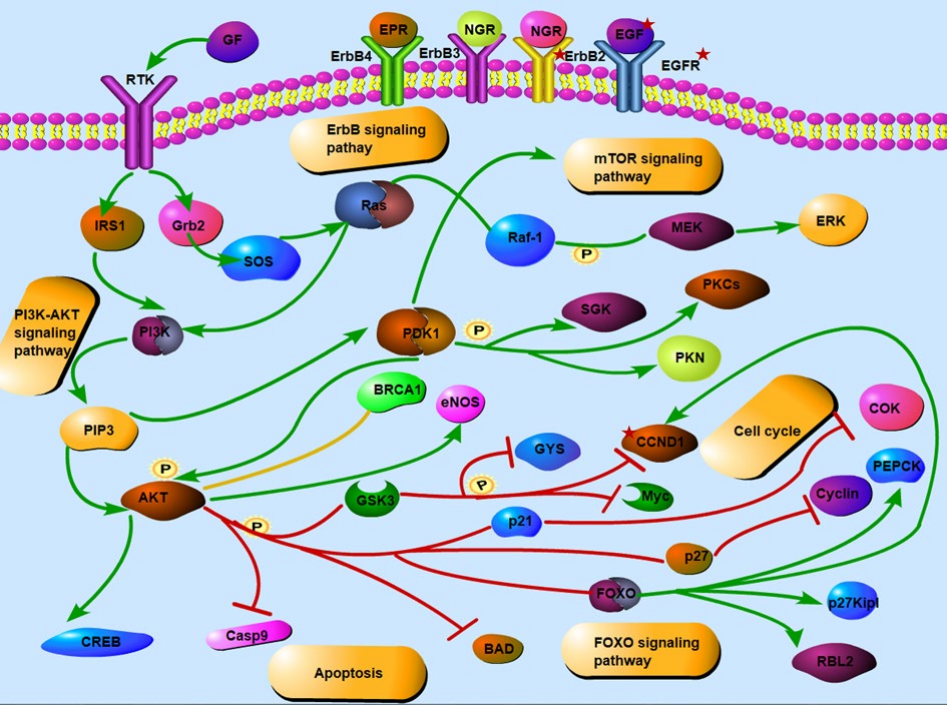
Regulatory pathways are mainly involved in the CKI treatment of ESCA. Green arrows indicate activation, red indicates inhibition, and yellow lines denote binding. P represents phosphorylation.

To clarify the multiple mechanisms of CKI on ESCA on a systematic level, we performed GO enrichment analysis including the biological process (BP), molecular function (MF), and cellular component (CC) and KEGG functional enrichment analysis of the selected PPI targets. Eventually, 559 enriched GO terms were identified, of which 477 were BPs, 54 were MFs, and 27 were CCs (FDR < 0.01 and *P* < 0.01). Figure 8A shows the top ten entries for BP, MF and CC, most of which were related to the cell cycle. To gain insights into the pharmacological mechanisms of CKI on ESCA, we performed KEGG analysis. The results demonstrated that 92 entries satisfy FDR < 0.01 and *P* < 0.01. Moreover, these targets were significantly enriched in many pathways related to cancer and signalling pathways, such as the PI3K-Akt signalling pathway, ErbB signalling pathway and FoxO signalling pathway (Figs. 8B, 9).

### Molecular docking verification

**Figure 10:**
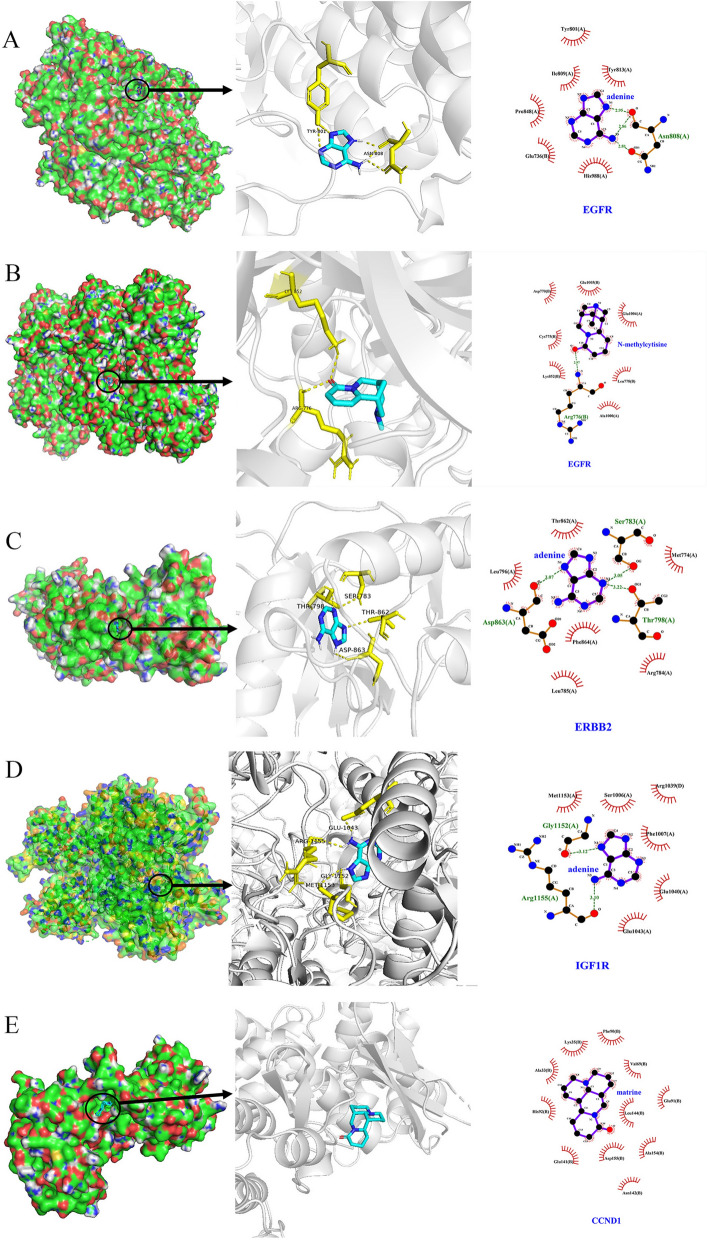
Molecular docking of the hub gene with its corresponding component. (**A**) EGFR with adenine; (**B**) EGFR with N-methylcytisine; (**C**) ErbB2 with adenine; (**D**) IGF1R with adenine; (**E**) CCND1 with matrine.

Four of the hub genes (EGFR, ErbB2, CCND1 and IGF1R) were directly related to the CKI active ingredients (adenine, N-methylcytisine and matrine). These 4 potential target proteins and their corresponding small-molecule ligand components were docked by AutoDock Vina. The docking results showed that the binding affinity was not greater than −6.0 kcal/mol, which proved that CKI components had good binding ability to the targets (Table 2). Figure 10 illustrates the interaction of the target compounds of the docking simulation. Adenine mainly forms two hydrogen bonds with the TYR-801 and ASN-808 residues on the EGFR protein, and a total of 6 residues are bound to the protein by hydrophobic interaction. In addition, the same EGFR protein formed two hydrogen bonds with residues LYS-852 and ARG-776 in addition to 7 hydrophobic bonds. Adenine formed 4 hydrogen bonds and 6 hydrophobic bonds with the ERBB2 protein and IGF1R protein, respectively, which proved that they are relatively tightly bound. Finally, although matrine does not form a hydrogen bond with CCND1, it binds to 10 residues of the protein by hydrophobic interaction.

**Table 2:** Molecular docking information.

No	Protein name	PDB ID	Protein structure	Test compounds	Affinity (kcal/mol)
1	EGFR	6DUK		Adenine	− 6.3
2	EGFR	6DUK		N-methylcytisine	− 6.7
3	ErbB2	3PP0		Adenine	− 6.2
4	IGF1R	5HZN		Adenine	− 6.0
5	CCND1	6P8G		Matrine	− 6.1

### Survival analysis

**Figure 11:**
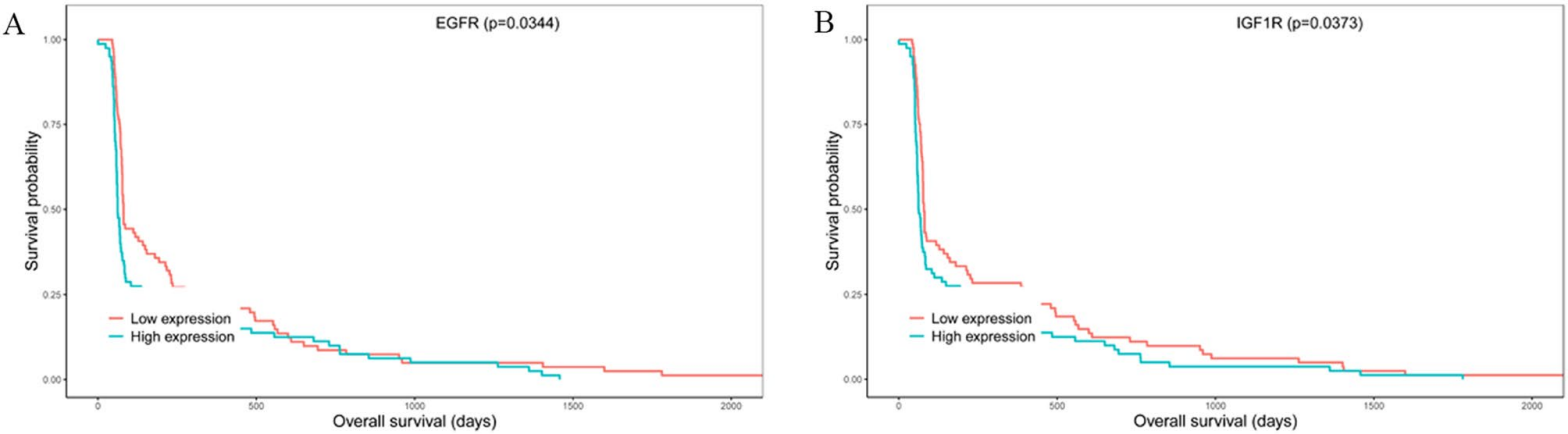
Survival analysis of hub genes. (**A**) EGFR; (**B**) IGF1R.

Kaplan–Meier survival analysis was performed to investigate overall survival. The results demonstrated that high expression of EGFR and IGF1R may be considered an effective prognostic indicator for ESCA patients (Fig. 11).

## Discussion

ESCA is one of the most common malignant tumours worldwide. With the development of medical technology, the treatment and prognosis of ESCA have improved, but it also poses a great threat to human life^24^. Although the pathogenesis of ESCA is still unclear, there are many causes, such as eating excessively hot food, smoking and drinking, obesity and gastroesophageal reflux disease^25,26^. ESCA also differs by region, sex, and race^27^. CKI has been proven to be immensely useful in the treatment of various cancers and also has relief and treatment effects for cancer pain^28^. According to real-world research in hospital systems, the application of CKI in the clinical treatment of ESCA is also famously used^10^.

In this study, we used integrated bioinformatics methods to explore the molecular mechanisms of CKI in the treatment of ESCA. WGCNA was employed to analyse key genes in ESCA and combined with network pharmacology to predict the therapeutic mechanism of CKI. Moreover, molecular docking methods were performed to verify the binding affinity of CKI with hub targets to validate the medicinal effects of CKI.

First, this study used the WGCNA method to explore the pathogenesis of ESCA and to study the link between gene expression data and clinical traits to screen for important gene modules. According to the WGCNA results, a total of 10 related modules were obtained, among which the blue module and the turquoise module were strongly related to multiple clinical traits, such as blue-new tumour event (*p* = 5e−04), blue-histological type (*p* = 2e−29), turquoise-cancer status (*p* = 2e−05) and turquoise-pathologic T (*p* = 0.008). Blue modules and turquoise modules were selected for network pharmacology analysis with the CKI predicted targets. Through network merging and PPI, a total of 16 gene targets were obtained with topological characteristics degree greater than the average. Among them, EGFR (degree = 21), ERBB2 (degree = 18), CCND1 (degree = 16) and IGF1R (degree = 15) were the predicted targets directly corresponding to the CKI components that were considered to be the hub genes related to CKI treatment of ESCA, and detailed discussions were conducted.

Epidermal growth receptor factor (EGFR) is an expression product of the proto-oncogene c-erb-B1 and is a member of subtype 1 of the receptor tyrosine kinase (RTK) family. The family also includes ErbB2, ErbB3 and ErbB4^29^. The binding of EGFR to a ligand activates intracellular tyrosine protein kinase activity, which phosphorylates the terminal tyrosine to activate downstream enzymes *and* initiate downstream signalling^30^. EGFR is overexpressed or mutated in most tumours, resulting in dysregulation of the signal transduction pathway, uncontrolled cell growth, and inhibition of cancer cell apoptosis^31^. Therefore, EGFR-targeted drugs are clinically used in a variety of cancers, and EGFR is also a hot target for tumour diagnosis and treatment. EGFR is highly expressed in both EA and ESCC. In addition, high expression of EGFR is closely related to the proliferation, infiltration and poor prognosis of ESCA cells^32–34^. Thus, abnormal EGFR expression is one of the serious pathogenic factors of ESCA. CKI can treat ESCA by regulating EGFR. ErbB2 is a transmembrane glycoprotein with protein tyrosine kinase (PTK) activity and a proto-oncogene of the human epidermal growth factor receptor family^35^. Overexpression of ErbB2 accelerates tumour growth, metastasis, and tumour blood vessel formation, increasing its invasion in vitro^36^. Equally important, ErbB2 can further improve the ability of tumour cells to migrate and adhere, promote tumour invasion and encourage local and/or distant metastasis^37^. Hoffmann^38^ proposed a diagnostic method to detect ErbB2 amplification in single disseminated cancer cells, demonstrating that ErbB2 amplification in esophageal cancer patients is significantly correlated with short-term survival. Previous studies have shown that ESCA can be treated by taking ErbB2 inhibitors such as trastuzumab and ramiximab^39^. The molecular docking results obtained in this study are similar to the docking results of the ErbB2 small-molecule antibody drugs erlotinib and lapatinib in the Rambabu Gundla study^40^. SER-783, THR-862, THR-798 and ASP-863 residues can all generate hydrogen bonds to connect with the compound. Therefore, we believe that CKI can treat ESCA by inhibiting ErbB2. CCND1 is a cell cycle regulating protein that can control the transition of the cell cycle from G1 to S phase, which is closely related to the occurrence and development of many tumours^41^. Matrine is one of the active components of CKI and has been shown to have anti-inflammatory, immunosuppressive, antitumour and antifibrotic effects^42^. Studies have shown that matrine can mediate the expression of CCND1 in breast cancer cells and thus inhibit cancer cells^43^. Guo^44^ detected rhabdomyosarcoma cells treated with matrine at different concentrations by MTT, flow cytometry, and RT-PCR and found that matrine significantly inhibits the proliferation of rhabdomyosarcoma cells by reducing the expression of CCND1 mRNA and blocking the cell cycle of the G0/G1 phase. Genome-wide screening revealed that the amplification of cyclin D1 is one of many genetic changes in ESCC. Accordingly, CKI may be used as an inhibitor of CCND1 to treat ESCA^45^. Insulin-like growth factor-1 receptor (IGF1R) is a tyrosine kinase that is involved in the pathogenesis of many cancers. After binding to the ligand, IGF1R can activate PI3K/AKT/mTOR and Ras/Raf/MEK/to activate the MAPK pathway, which can regulate cell proliferation, survival, differentiation, movement, invasion and angiogenesis^46,47^. Studies have shown that IGF1R is overexpressed in cancer tissues compared to normal tissues adjacent to the cancer. In addition, a mouse xenograft model was used to test the function of IGF-1R in vitro and in vivo. IGF1R was found to have carcinogenic effects in regulating cell proliferation, colony formation, the cell cycle and apoptosis^48,49^.

The enriched GO analysis indicated that the hub genes localized mainly to the cyclin-dependent protein kinase holoenzyme complex, protein kinase complex and serine/threonine protein kinase complex, while their molecular functions were associated with the cell cycle. Similarly, KEGG pathway analysis showed enrichment in some cancer pathways and signalling pathways, such as the PI3K-Akt signalling pathway and ErbB signalling pathway. The ErbB family, after binding to its corresponding ligands (EGF, TGF, AR, etc.), downstream related genes such as PI3K/AKT and MAPK can be activated, thereby regulating cell proliferation, differentiation, migration, and apoptosis activities^50^. Studies have shown that the PI3K/AKT pathway is abnormally activated in a variety of cancers, such as esophageal, gastric, and breast cancer^51–53^.

Upstream genes such as ErbB2 and various growth factors such as EGF and IGF1 can activate PI3K, resulting in aberrant activation of the PI3K/AKT pathway^54^. Additionally, the deviant activation of the PI3K/AKT pathway inhibits the degradation of CCND1, increases its expression, promotes its shift to the nucleus, and interferes with the transition from G1 to S phase of the cell cycle^55^. The ligands IGF-1 and IGF-2 bind to IGF1R, leading to receptor autophosphorylation and the activation of various signalling pathways, including the PI3K/AKT pathway, which leads to cell proliferation and prevents apoptosis^56^. Abnormal activation of pathways caused by abnormal expression of these proteins is a critical factor affecting the progression of esophageal cancer. Zhang^57^ found that CKI can increase the ability to inhibit lung cancer cell proliferation and increase sensitivity to gefitinib by downregulating the PI3K/AKT pathway. A study has also shown that matrine derivatives, which are one of the main components of CKI, can be downregulated, CCND1, and attenuated the PI3K/Akt pathway to induce G1 cell cycle arrest and autophagy in cancer cells through immunofluorescence analysis, western blotting and murine models^58^. Therefore, these potential targets and pathways may be the key to the CKI treatment of ESCA.

The central idea of TCM has a lot in common with network pharmacology, which can explain the treatment process of many complex diseases in a system manner^59^. In previous studies, Li^60^ provided a powerful means for identifying mechanisms of Ge–Gen–Qin–Lian decoction in the treatment of type 2 diabetes through the network pharmacology strategy. Liang^61^ exploited drugCIPHER to incorporate the traditional network pharmacology concept to analyse the target network of the TCM traditional prescription Liu-Wei-Di-Huang pill. This study was based on the network pharmacology method combined with WGCNA analysis, aiming to accurately detect the genes related to ESCA from the aspect of close to clinical traits to analyse the mechanism of CKI treatment of ESCA. However, there are some limitations in this method. First, our data collection is based on existing database information, so it may produce deviations and incomplete results. Second, biological experiments are urgently needed to validate our results because our study was performed based on data analysis.

## Conclusion

In summary, by combining WGCNA and a network pharmacology method, we revealed that CKI controlled the growth of ESCA by regulating potential hub genes, such as EGFR, ErbB2, CCND1 and IGF1R, as well as important related pathways. The study preliminarily verified and predicted the molecular mechanism of CKI against ESCA but still needs further experimental verification. These findings provide insights into the underlying mechanism of CKI for the treatment of ESCA and provide a reference for the study of the more complex mechanism of action of this Chinese herbal compound.

## Methods

### Data collection and preprocessing

RNA sequencing data in fragments per kilobase million (FPKM) of ESCA were obtained from the TCGA data portal (https://portal.gdc.cancer.gov) in September 2019, with a total of 164 samples. The clinical metadata of the 164 samples were also downloaded and filtered for useful information. After the removal of samples containing incomplete analytical data and/or other malignancies, 161 samples were retained. Since some genes lacked significant changes in expression between samples, we chose the top 5,000 genes that were most important in terms of differential expression for the next WGCNA analysis.

### Weighted gene co-expression network analysis and module preservation

The gene co-expression networks were constructed by the WGCNA package. We used the similarity between gene expression profiles to construct a similarity matrix based on pairwise Pearson correlation coefficient matrices. The similarity matrix was transformed into an adjacency matrix using a power adjacency function^18,62^. The appropriate soft threshold power β was selected by using the integration function (pickSoftThresshold) in the WGCNA software package. With this soft threshold function, the co-expression similarity was improved to achieve a scale-free topology^63,64^. Then, we reconstructed the topological overlap matrix by calculating the topological overlap measure (TOM), which is a robust measure of network interconnectedness^65,66^. The dynamic tree-cut algorithm method was adopted to identify the module of gene co-expression with the maxBlockSize of 6,000, minModuleSize of 30 and mergeCutHeight of 0.2.

### Identification of clinically significant modules

Module eigengene (ME) is the first principal component of each gene module, and the expression of ME is considered representative of all genes in one module. The module membership (MM) is the correlation between the ME and the gene expression profile. Gene Significance (GS) is the absolute value of the correlation between a specific gene and a clinical trait. According to ME, GS, and MM, we can associate modules with clinical traits not only to calculate the correlation between ME and clinical traits but also to analyse clinically vital modules^18^.

### Construction of predictive target network for CKI components

The 3D chemical structure data of 16 active ingredients were imported into the Search Tool for Interactions of Chemicals (STITCH)^67^, SuperPred^68^, SwissTargetPrediction^69^ and Traditional Chinese Medicine Systems Pharmacology Database and Analysis Platform (TCMSP) ^70^ databases for retrieval. The predicted multiple target information of the compounds and the obtained information were introduced into Cytoscape 3.6.1 (https://www.cytoscape.org/) to obtain a compound-predicted target network map. Cytoscape is bioinformatics analysis software that visualizes biological pathways and intermolecular interaction networks and provides a basic set of data integration, analysis and visualization capabilities for complex network analyses^71^.

### Network construction and analysis of CKI in the treatment of ESCA

The compound-predicted target network and clinically important module network obtained from WGCNA analysis were merged in Cytoscape. The overlapping proteins in the two networks are likely to be potential targets for the treatment of ESCA by the active ingredients of CKI. The Search Tool for the Retrieval of Interacting Genes/Proteins (STRING) 10.5 (https://string-db.org/) is a database of known and predicted protein interactions that contains direct and indirect protein associations^72^. The overlapping genes were input into the STRING 10.5 database, and the species selection "Homo sapiens" was selected as the confidence data with a scoring value greater than 0.7 and 1_st_ shell no more than 20. Then, the data were introduced into Cytoscape to construct a protein–protein interaction (PPI) network.

### Gene ontology (GO) functional and kyoto encyclopedia of genes and genomes (KEGG) pathway enrichment analysis

The GO database (https://geneontology.org/) was used to identify the possible biological mechanisms using high-throughput genome or transcriptome data^73^. KEGG is a reference knowledge base for biological interpretation of genome sequences and other high-throughput data, which is a reference for biological interpretation of genome sequences and other high-throughput data^74^. In addition, the R package clusterProfiler was used to perform GO and KEGG functional enrichment analysis^75^.

### Molecular docking simulation

Molecular docking can reflect the binding energetics of drug molecules to protein receptors by calculating the binding affinity between ligands and receptors and the corresponding intermolecular interactions^76,77^. The potential targets that were directly related to the CKI active components were imported into the Protein Data Bank (PDB) (https://www.rcsb.org/) database to find their 3D structure^78^. Proteins that met the following conditions were considered appropriate protein conformations: (1) The 3D protein structures were determined by X-ray crystallography. (2) Crystal resolution Protein was less than 3 Å; (3) Genotype protein analysis was reliable. Molecular docking simulation of potential targets and their corresponding components was performed using AutoDock 4.2 and AutoDock Vina software according to published methods^79,80^. A suitable grid box size with a spacing of 1.0 Å between grid points was generated to cover almost the entire favourable protein binding site. The X, Y and Z centres are adjusted according to different macromolecular forms. The results of docking are displayed by Pymol and Ligplot^81^.

### Survival analysis of hub genes

Survival analysis was conducted using the Kaplan–Meier method and log-rank tests. Hazard ratios (HRs) were calculated using a Cox proportional hazards model with R software and the “survival” R package. In the survival analysis, death from any cause was considered an event^82^.

### Ethics approval and consent to participate

Ethical approval was not necessary in the current study because our study gathered data from TCGA, and this procedure did not address any patients’ personal data or harm any patient.

## Supplementary information

Supplementary file1

Supplementary file2

Supplementary file3

## Data Availability

CKI compounds, cut off 0.1 blue network nodes and cut off 0.1 turquoise network nodes are available in the Supplementary Source files.
