# Supplementary material for: Integrated bioinformatics analysis to decipher molecular mechanism of compound Kushen injection for esophageal cancer by combining WGCNA with network pharmacology
Source: Sci Rep. 2020 Jul 29;10:12745. doi: 10.1038/s41598-020-69708-2 (PMC7391752; doi:10.1038/s41598-020-69708-2)
Supplement: Supplementary file 1 — Supplementary file1 [file 41598_2020_69708_MOESM1_ESM.docx]

**Integrated Bioinformatics Analysis to Decipher Molecular Mechanism of Compound Kushen Injection for Esophageal Cancer by Combining WGCNA with Network Pharmacology**

**Wei Zhou^1^, Jiarui Wu^1^*****, Jingyuan Zhang^1^, Xinkui Liu^1^, Siyu Guo^1^, ShanShan Jia^1^, Xiaomeng Zhang^1^, Yingli Zhu^1^, Miaomiao Wang^1^**

1Beijing University of Chinese Medicine, Beijing, 100102, China

* Corresponding email: exogamy@163.com

| nodeName | altName | nodeAttr[nodesPresent, ] |
| --- | --- | --- |
| MUC17 | protein_coding | turquoise |
| REG4 | protein_coding | turquoise |
| MUC13 | protein_coding | turquoise |
| OLFM4 | protein_coding | turquoise |
| CTSE | protein_coding | turquoise |
| REG1A | protein_coding | turquoise |
| DMBT1 | protein_coding | turquoise |
| VIL1 | protein_coding | turquoise |
| EPS8L3 | protein_coding | turquoise |
| LGALS4 | protein_coding | turquoise |
| TSPAN8 | protein_coding | turquoise |
| TFF1 | protein_coding | turquoise |
| HNF4A | protein_coding | turquoise |
| MGAM2 | protein_coding | turquoise |
| SPINK1 | protein_coding | turquoise |
| GJB1 | protein_coding | turquoise |
| PIGR | protein_coding | turquoise |
| HMGCS2 | protein_coding | turquoise |
| AGR3 | protein_coding | turquoise |
| MUC5B | protein_coding | turquoise |
| HNF1A | protein_coding | turquoise |
| MUC5AC | protein_coding | turquoise |
| ANKS4B | protein_coding | turquoise |
| CAPN8 | protein_coding | turquoise |
| PPP1R1B | protein_coding | turquoise |
| MUC3A | protein_coding | turquoise |
| SLC6A15 | protein_coding | turquoise |
| BPIFB1 | protein_coding | turquoise |
| CDH17 | protein_coding | turquoise |
| ERN2 | protein_coding | turquoise |
| USH1C | protein_coding | turquoise |
| FOXA3 | protein_coding | turquoise |
| SLC44A4 | protein_coding | turquoise |
| CLDN18 | protein_coding | turquoise |
| PDX1 | protein_coding | turquoise |
| CFTR | protein_coding | turquoise |
| MYO1A | protein_coding | turquoise |
| HOXD11 | protein_coding | turquoise |
| PROM1 | protein_coding | turquoise |
| DPCR1 | protein_coding | turquoise |
| TRIM15 | protein_coding | turquoise |
| TRIM31 | protein_coding | turquoise |
| HGD | protein_coding | turquoise |
| CLDN3 | protein_coding | turquoise |
| AGR2 | protein_coding | turquoise |
| PLA2G2A | protein_coding | turquoise |
| TMC5 | protein_coding | turquoise |
| BCL2L15 | protein_coding | turquoise |
| HKDC1 | protein_coding | turquoise |
| SLC6A20 | protein_coding | turquoise |
| SMIM24 | protein_coding | turquoise |
| FOXA2 | protein_coding | turquoise |
| DDC | protein_coding | turquoise |
| SPDEF | protein_coding | turquoise |
| AOC1 | protein_coding | turquoise |
| SULT1C2 | protein_coding | turquoise |
| MUC6 | protein_coding | turquoise |
| CDX2 | protein_coding | turquoise |
| MYO7B | protein_coding | turquoise |
| MUC2 | long_non_coding | turquoise |
| PLEKHS1 | protein_coding | turquoise |
| HOXD10 | protein_coding | turquoise |
| C9orf152 | protein_coding | turquoise |
| TOX3 | protein_coding | turquoise |
| ADH1C | protein_coding | turquoise |
| CDHR5 | protein_coding | turquoise |
| SLC34A2 | protein_coding | turquoise |
| HS6ST2 | protein_coding | turquoise |
| GCNT3 | protein_coding | turquoise |
| PRR15L | protein_coding | turquoise |
| SYT13 | protein_coding | turquoise |
| BCAS1 | protein_coding | turquoise |
| AZGP1 | protein_coding | turquoise |
| C2orf72 | protein_coding | turquoise |
| PRAP1 | protein_coding | turquoise |
| DLX6-AS1 | long_non_coding | turquoise |
| TMPRSS3 | protein_coding | turquoise |
| ARL14 | protein_coding | turquoise |
| RBP4 | protein_coding | turquoise |
| KRT7 | protein_coding | turquoise |
| AQP5 | protein_coding | turquoise |
| PIP5K1B | protein_coding | turquoise |
| KLHDC7A | protein_coding | turquoise |
| GOLT1A | protein_coding | turquoise |
| MYRF | protein_coding | turquoise |
| VNN1 | protein_coding | turquoise |
| CNTN1 | protein_coding | turquoise |
| FAM3B | protein_coding | turquoise |
| RFLNA | protein_coding | turquoise |
| SGK2 | protein_coding | turquoise |
| MSLN | protein_coding | turquoise |
| LINC01980 | long_non_coding | turquoise |
| VSIG2 | protein_coding | turquoise |
| NTRK2 | protein_coding | turquoise |
| DLX5 | protein_coding | turquoise |
| SHH | protein_coding | turquoise |
| HNF4G | protein_coding | turquoise |
| CA8 | protein_coding | turquoise |
| RIMS2 | protein_coding | turquoise |
| TFF3 | protein_coding | turquoise |
| ANXA10 | protein_coding | turquoise |
| GAL3ST1 | protein_coding | turquoise |
| LINC00958 | long_non_coding | turquoise |
| PLEKHG4B | protein_coding | turquoise |
| GPR27 | protein_coding | turquoise |
| TMEM139 | protein_coding | turquoise |
| TESC | protein_coding | turquoise |
| KCNQ5 | protein_coding | turquoise |
| CLDN2 | protein_coding | turquoise |
| ZG16B | protein_coding | turquoise |
| GPR35 | protein_coding | turquoise |
| FSTL4 | protein_coding | turquoise |
| ARHGEF38 | protein_coding | turquoise |
| KIAA1324 | protein_coding | turquoise |
| GATA4 | protein_coding | turquoise |
| KLRG2 | protein_coding | turquoise |
| ARSE | protein_coding | turquoise |
| TLX1 | protein_coding | turquoise |
| MLPH | protein_coding | turquoise |
| SULT1B1 | protein_coding | turquoise |
| SLC4A4 | protein_coding | turquoise |
| CYP24A1 | protein_coding | turquoise |
| PANX2 | protein_coding | turquoise |
| AKR7A3 | protein_coding | turquoise |
| SLCO1A2 | protein_coding | turquoise |
| F5 | protein_coding | turquoise |
| HOXA13 | protein_coding | turquoise |
| TMPRSS2 | protein_coding | turquoise |
| CDHR2 | protein_coding | turquoise |
| CYP3A5 | protein_coding | turquoise |
| BEX2 | protein_coding | turquoise |
| B3GALT5 | protein_coding | turquoise |
| KIF12 | protein_coding | turquoise |
| MMP13 | protein_coding | turquoise |
| DUSP9 | protein_coding | turquoise |
| B4GALNT4 | protein_coding | turquoise |
| PDE11A | protein_coding | turquoise |
| METTL7B | protein_coding | turquoise |
| PRR26 | protein_coding | turquoise |
| GNG4 | protein_coding | turquoise |
| SOWAHA | protein_coding | turquoise |
| PTPRH | protein_coding | turquoise |
| UCA1 | long_non_coding | turquoise |
| RORC | protein_coding | turquoise |
| CYP27C1 | protein_coding | turquoise |
| LYZ | protein_coding | turquoise |
| BCL2L14 | protein_coding | turquoise |
| NXPH4 | protein_coding | turquoise |
| SEMA4G | protein_coding | turquoise |
| AGT | protein_coding | turquoise |
| UNC5CL | protein_coding | turquoise |
| LY6K | protein_coding | turquoise |
| TNNT1 | protein_coding | turquoise |
| PLEKHB1 | protein_coding | turquoise |
| NPR3 | protein_coding | turquoise |
| ONECUT2 | protein_coding | turquoise |
| GDF15 | protein_coding | turquoise |
| DNAJC22 | protein_coding | turquoise |
| GATA6 | protein_coding | turquoise |
| RNF165 | protein_coding | turquoise |
| GDA | protein_coding | turquoise |
| UCHL1 | protein_coding | turquoise |
| CXCL3 | protein_coding | turquoise |
| PRSS3 | protein_coding | turquoise |
| EPHA10 | protein_coding | turquoise |
| CAPN13 | protein_coding | turquoise |
| NCMAP | protein_coding | turquoise |
| HSD17B2 | protein_coding | turquoise |
| SERPINA1 | protein_coding | turquoise |
| SEMA3D | protein_coding | turquoise |
| KCNE3 | protein_coding | turquoise |
| TSPAN1 | protein_coding | turquoise |
| PITX2 | protein_coding | turquoise |
| FA2H | protein_coding | turquoise |
| NGFR | protein_coding | turquoise |
| C4orf19 | protein_coding | turquoise |
| C6orf223 | protein_coding | turquoise |
| KCNG1 | protein_coding | turquoise |
| RHPN2 | protein_coding | turquoise |
| MISP | protein_coding | turquoise |
| SLITRK6 | protein_coding | turquoise |
| PLAC8 | protein_coding | turquoise |
| PKDCC | protein_coding | turquoise |
| NELL2 | protein_coding | turquoise |
| WNK4 | protein_coding | turquoise |
| BCHE | protein_coding | turquoise |
| PLEKHA6 | protein_coding | turquoise |
| CCNI2 | protein_coding | turquoise |
| CPS1 | protein_coding | turquoise |
| RYR1 | protein_coding | turquoise |
| C16orf74 | protein_coding | turquoise |
| PRR15 | protein_coding | turquoise |
| KCNJ15 | protein_coding | turquoise |
| SAMD5 | protein_coding | turquoise |
| AL365181.2 | long_non_coding | turquoise |
| FGFR4 | protein_coding | turquoise |
| B4GALNT1 | protein_coding | turquoise |
| EDARADD | protein_coding | turquoise |
| C2CD4A | protein_coding | turquoise |
| UBXN10 | protein_coding | turquoise |
| ADAM23 | protein_coding | turquoise |
| HOXB13 | protein_coding | turquoise |
| HOXB-AS3 | long_non_coding | turquoise |
| CAPN5 | protein_coding | turquoise |
| SLC17A9 | protein_coding | turquoise |
| SLC5A1 | protein_coding | turquoise |
| HID1 | protein_coding | turquoise |
| FST | protein_coding | turquoise |
| AL513318.2 | long_non_coding | turquoise |
| ACSL5 | protein_coding | turquoise |
| GABRA3 | protein_coding | turquoise |
| LTK | protein_coding | turquoise |
| HAP1 | protein_coding | turquoise |
| NOS2 | protein_coding | turquoise |
| C2CD4B | protein_coding | turquoise |
| HTR1D | protein_coding | turquoise |
| TBX18 | protein_coding | turquoise |
| SMPD3 | protein_coding | turquoise |
| CLIC6 | protein_coding | turquoise |
| RTN4RL1 | protein_coding | turquoise |
| AKR7L | pseudogene | turquoise |
| CREB3L1 | protein_coding | turquoise |
| TRABD2A | protein_coding | turquoise |
| TBX1 | protein_coding | turquoise |
| FCGBP | protein_coding | turquoise |
| CES3 | protein_coding | turquoise |
| GPRC5A | protein_coding | turquoise |
| RANBP17 | protein_coding | turquoise |
| TNFRSF14-AS1 | long_non_coding | turquoise |
| ADRA2A | protein_coding | turquoise |
| PRDM16 | protein_coding | turquoise |
| NPC1L1 | protein_coding | turquoise |
| KCNK5 | protein_coding | turquoise |
| COL4A6 | protein_coding | turquoise |
| ZNF114 | protein_coding | turquoise |
| PTPRN2 | protein_coding | turquoise |
| MUC1 | protein_coding | turquoise |
| PCDH19 | protein_coding | turquoise |
| TJP3 | protein_coding | turquoise |
| GPNMB | protein_coding | turquoise |
| SLC52A1 | protein_coding | turquoise |
| RBP1 | protein_coding | turquoise |
| SNAI2 | protein_coding | turquoise |
| CLIC5 | protein_coding | turquoise |
| FSCN1 | protein_coding | turquoise |
| MUC12 | protein_coding | turquoise |
| PRIMA1 | protein_coding | turquoise |
| PDK4 | protein_coding | turquoise |
| CYP2W1 | protein_coding | turquoise |
| LONRF3 | protein_coding | turquoise |
| CACNA1D | protein_coding | turquoise |
| ANKRD18B | protein_coding | turquoise |
| ARTN | protein_coding | turquoise |
| GALNT14 | protein_coding | turquoise |
| CHDH | protein_coding | turquoise |
| SLC9A2 | protein_coding | turquoise |
| TENM3 | protein_coding | turquoise |
| CRACR2B | protein_coding | turquoise |
| SLC19A3 | protein_coding | turquoise |
| RAB17 | protein_coding | turquoise |
| PTPRR | protein_coding | turquoise |
| B3GNT3 | protein_coding | turquoise |
| ZNF69 | protein_coding | turquoise |
| IQCA1 | protein_coding | turquoise |
| PRLR | protein_coding | turquoise |
| SRMS | protein_coding | turquoise |
| KCTD14 | protein_coding | turquoise |
| UBD | protein_coding | turquoise |
| ZBED9 | protein_coding | turquoise |
| CECR2 | protein_coding | turquoise |
| IQGAP2 | protein_coding | turquoise |
| DEGS2 | protein_coding | turquoise |
| WDR66 | protein_coding | turquoise |
| ARFGEF3 | protein_coding | turquoise |
| SMPDL3B | protein_coding | turquoise |
| TMEM150B | protein_coding | turquoise |
| ASPHD1 | protein_coding | turquoise |
| IL17RB | protein_coding | turquoise |
| MNX1 | protein_coding | turquoise |
| RNF183 | protein_coding | turquoise |
| DPP4 | protein_coding | turquoise |
| FZD10-AS1 | long_non_coding | turquoise |
| HOXB6 | protein_coding | turquoise |
| PDE4C | protein_coding | turquoise |
| TNC | protein_coding | turquoise |
| STXBP6 | protein_coding | turquoise |
| TUSC3 | protein_coding | turquoise |
| CGN | protein_coding | turquoise |
| LINC02086 | long_non_coding | turquoise |
| PLA2G16 | protein_coding | turquoise |
| TNFRSF11B | protein_coding | turquoise |
| MGAT3 | protein_coding | turquoise |
| ACHE | protein_coding | turquoise |
| PPARG | protein_coding | turquoise |
| DUSP4 | protein_coding | turquoise |
| KREMEN2 | protein_coding | turquoise |
| B3GNT7 | protein_coding | turquoise |
| SLCO4A1-AS1 | long_non_coding | turquoise |
| NR5A2 | protein_coding | turquoise |
| SHOX2 | protein_coding | turquoise |
| SCIN | protein_coding | turquoise |
| PLS1 | protein_coding | turquoise |
| HOXB9 | protein_coding | turquoise |
| TOR4A | protein_coding | turquoise |
| PART1 | long_non_coding | turquoise |
| GALNT5 | protein_coding | turquoise |
| CLCN4 | protein_coding | turquoise |
| MLF1 | protein_coding | turquoise |
| ARSI | protein_coding | turquoise |
| SYTL2 | protein_coding | turquoise |
| GAMT | protein_coding | turquoise |
| IRF8 | protein_coding | turquoise |
| GUCY2C | protein_coding | turquoise |
| KCNK15 | protein_coding | turquoise |
| FER1L4 | pseudogene | turquoise |
| OSBPL6 | protein_coding | turquoise |
| SELENBP1 | protein_coding | turquoise |
| KIAA1211 | protein_coding | turquoise |
| NR3C2 | protein_coding | turquoise |
| FBP1 | protein_coding | turquoise |
| CXCL2 | protein_coding | turquoise |
| GLI3 | protein_coding | turquoise |
| GIPC2 | protein_coding | turquoise |
| FUT4 | protein_coding | turquoise |
| ADGRV1 | protein_coding | turquoise |
| AC009065.5 | long_non_coding | turquoise |
| SMIM22 | protein_coding | turquoise |
| VILL | protein_coding | turquoise |
| MLXIPL | protein_coding | turquoise |
| AL365181.3 | long_non_coding | turquoise |
| SYNE4 | protein_coding | turquoise |
| HPGD | protein_coding | turquoise |
| CDC42EP5 | protein_coding | turquoise |
| BCO1 | protein_coding | turquoise |
| GJB7 | protein_coding | turquoise |
| TRPA1 | protein_coding | turquoise |
| FOXP2 | protein_coding | turquoise |
| KBTBD11 | protein_coding | turquoise |
| MYB | protein_coding | turquoise |
| PAQR8 | protein_coding | turquoise |
| ADAM28 | protein_coding | turquoise |
| NTF4 | protein_coding | turquoise |
| PLCH1 | protein_coding | turquoise |
| NOSTRIN | protein_coding | turquoise |
| XKR9 | protein_coding | turquoise |
| LAMA1 | protein_coding | turquoise |
| SDK1 | protein_coding | turquoise |
| BNIP3 | protein_coding | turquoise |
| ICAM5 | protein_coding | turquoise |
| XK | protein_coding | turquoise |
| AC009065.2 | long_non_coding | turquoise |
| DDAH1 | protein_coding | turquoise |
| ANKRD65 | protein_coding | turquoise |
| VWA2 | protein_coding | turquoise |
| ETNK2 | protein_coding | turquoise |
| ADD2 | protein_coding | turquoise |
| AC091563.1 | long_non_coding | turquoise |
| DACH1 | protein_coding | turquoise |
| CFB | protein_coding | turquoise |
| ALDH3B1 | protein_coding | turquoise |
| FOXC2 | protein_coding | turquoise |
| EXOC3L4 | protein_coding | turquoise |
| COL7A1 | protein_coding | turquoise |
| VANGL2 | protein_coding | turquoise |
| PROX1 | protein_coding | turquoise |
| HOXA7 | protein_coding | turquoise |
| UCN2 | protein_coding | turquoise |
| SLC43A1 | protein_coding | turquoise |
| GPR160 | protein_coding | turquoise |
| ABCC3 | protein_coding | turquoise |
| GSDMB | protein_coding | turquoise |
| MYO15B | protein_coding | turquoise |
| FMO5 | protein_coding | turquoise |
| CORO6 | protein_coding | turquoise |
| ALOX12P2 | pseudogene | turquoise |
| HOXB5 | protein_coding | turquoise |
| SLAIN1 | protein_coding | turquoise |
| LINC00857 | long_non_coding | turquoise |
| PIWIL4 | protein_coding | turquoise |
| SIX1 | protein_coding | turquoise |
| C2orf70 | protein_coding | turquoise |
| PNCK | protein_coding | turquoise |
| KLK1 | protein_coding | turquoise |
| TDRP | protein_coding | turquoise |
| TMEM163 | protein_coding | turquoise |
| FLRT2 | protein_coding | turquoise |
| MFSD4A | protein_coding | turquoise |
| MYEOV | protein_coding | turquoise |
| KCNN4 | protein_coding | turquoise |
| RNLS | protein_coding | turquoise |
| TRPV4 | protein_coding | turquoise |
| SMO | protein_coding | turquoise |
| COL4A5 | protein_coding | turquoise |
| TSPAN12 | protein_coding | turquoise |
| ACY3 | protein_coding | turquoise |
| KCNH8 | protein_coding | turquoise |
| AGMAT | protein_coding | turquoise |
| KCNQ1 | protein_coding | turquoise |
| FRMD5 | protein_coding | turquoise |
| BAIAP2L2 | protein_coding | turquoise |
| PTPRS | protein_coding | turquoise |
| KIAA1549L | protein_coding | turquoise |
| CCDC68 | protein_coding | turquoise |
| MN1 | protein_coding | turquoise |
| GJA1 | protein_coding | turquoise |
| ATP8A1 | protein_coding | turquoise |
| FBXO27 | protein_coding | turquoise |
| DNAH6 | protein_coding | turquoise |
| ACSM3 | protein_coding | turquoise |
| ISM1 | protein_coding | turquoise |
| RADIL | protein_coding | turquoise |
| WNT9A | protein_coding | turquoise |
| SLC7A8 | protein_coding | turquoise |
| GCNT1 | protein_coding | turquoise |
| SH3BGRL2 | protein_coding | turquoise |
| NLGN4X | protein_coding | turquoise |
| COPZ2 | protein_coding | turquoise |
| SGSM1 | protein_coding | turquoise |
| ZFHX4 | protein_coding | turquoise |
| EPCAM | protein_coding | turquoise |
| RND1 | protein_coding | turquoise |
| ATP2A3 | protein_coding | turquoise |
| HOXA11 | protein_coding | turquoise |
| GRIN2D | protein_coding | turquoise |
| RIMKLA | protein_coding | turquoise |
| LRP12 | protein_coding | turquoise |
| EPHB6 | protein_coding | turquoise |
| SLC40A1 | protein_coding | turquoise |
| KRT8P3 | pseudogene | turquoise |
| ASRGL1 | protein_coding | turquoise |
| SLC27A2 | protein_coding | turquoise |
| DEPTOR | protein_coding | turquoise |
| AC136475.3 | long_non_coding | turquoise |
| KCNMB2-AS1 | long_non_coding | turquoise |
| GATM | protein_coding | turquoise |
| KRT8P45 | pseudogene | turquoise |
| AMN | protein_coding | turquoise |
| CLMN | protein_coding | turquoise |
| ADAP1 | protein_coding | turquoise |
| CELSR3 | protein_coding | turquoise |
| SEMA3B | protein_coding | turquoise |
| RNF128 | protein_coding | turquoise |
| MACC1 | protein_coding | turquoise |
| TRIM9 | protein_coding | turquoise |
| DAPK1 | protein_coding | turquoise |
| SORBS2 | protein_coding | turquoise |
| MMP17 | protein_coding | turquoise |
| GGT1 | protein_coding | turquoise |
| AC004982.2 | long_non_coding | turquoise |
| AC022075.1 | long_non_coding | turquoise |
| RNF157 | protein_coding | turquoise |
| GRAMD1B | protein_coding | turquoise |
| SLC41A2 | protein_coding | turquoise |
| BEND7 | protein_coding | turquoise |
| ICA1 | protein_coding | turquoise |
| ATP7B | protein_coding | turquoise |
| DFNA5 | protein_coding | turquoise |
| EVC2 | protein_coding | turquoise |
| LINC00884 | long_non_coding | turquoise |
| DYNC1I1 | protein_coding | turquoise |
| TP73 | protein_coding | turquoise |
| GLB1L2 | protein_coding | turquoise |
| SYBU | protein_coding | turquoise |
| DNALI1 | protein_coding | turquoise |
| PHLDB2 | protein_coding | turquoise |
| CNTNAP3B | protein_coding | turquoise |
| ANG | protein_coding | turquoise |
| ANKRD33B | protein_coding | turquoise |
| THNSL2 | protein_coding | turquoise |
| CHN2 | protein_coding | turquoise |
| KRT8 | protein_coding | turquoise |
| DOK7 | protein_coding | turquoise |
| HEPH | protein_coding | turquoise |
| TMEM45A | protein_coding | turquoise |
| AL590666.2 | long_non_coding | turquoise |
| RARRES1 | protein_coding | turquoise |
| IGSF9B | protein_coding | turquoise |
| SIDT1 | protein_coding | turquoise |
| KLHL13 | protein_coding | turquoise |
| CRLF1 | protein_coding | turquoise |
| SLC1A1 | protein_coding | turquoise |
| TMEM92 | protein_coding | turquoise |
| FHOD3 | protein_coding | turquoise |
| C15orf59 | protein_coding | turquoise |
| VSIG1 | protein_coding | turquoise |
| STC2 | protein_coding | turquoise |
| EFNB3 | protein_coding | turquoise |
| SLC25A25-AS1 | long_non_coding | turquoise |
| REPS2 | protein_coding | turquoise |
| GHR | protein_coding | turquoise |
| MFAP2 | protein_coding | turquoise |
| CLDN23 | protein_coding | turquoise |
| TNFRSF11A | protein_coding | turquoise |
| TMEM125 | protein_coding | turquoise |
| BIRC3 | protein_coding | turquoise |
| STAC | protein_coding | turquoise |
| AMOT | protein_coding | turquoise |
| H2AFY2 | protein_coding | turquoise |
| PTGER2 | protein_coding | turquoise |
| TNIK | protein_coding | turquoise |
| GBP1P1 | pseudogene | turquoise |
| CCRL2 | protein_coding | turquoise |
| RAB19 | protein_coding | turquoise |
| NECTIN3 | protein_coding | turquoise |
| GOLM1 | protein_coding | turquoise |
| SLC44A3 | protein_coding | turquoise |
| FAM171A2 | protein_coding | turquoise |
| SHROOM3 | protein_coding | turquoise |
| CRYM | protein_coding | turquoise |
| TRNP1 | protein_coding | turquoise |
| ENPP4 | protein_coding | turquoise |
| AC124798.1 | long_non_coding | turquoise |
| NCR3LG1 | protein_coding | turquoise |
| BEND6 | protein_coding | turquoise |
| CYSTM1 | protein_coding | turquoise |
| NAV3 | protein_coding | turquoise |
| MAGEE1 | protein_coding | turquoise |
| PPM1H | protein_coding | turquoise |
| CCL28 | protein_coding | turquoise |
| FNDC10 | protein_coding | turquoise |
| SERPINE2 | protein_coding | turquoise |
| CNTNAP3 | protein_coding | turquoise |
| LGALS2 | protein_coding | turquoise |
| CAMK2N1 | protein_coding | turquoise |
| PPFIA4 | protein_coding | turquoise |
| BTNL9 | protein_coding | turquoise |
| ZC3H12B | protein_coding | turquoise |
| SIM2 | protein_coding | turquoise |
| HOMER3 | protein_coding | turquoise |
| AC103702.2 | long_non_coding | turquoise |
| CLEC2B | protein_coding | turquoise |
| ENC1 | protein_coding | turquoise |
| GNGT1 | protein_coding | turquoise |
| RNASE4 | protein_coding | turquoise |
| SUGCT | protein_coding | turquoise |
| SLCO4A1 | protein_coding | turquoise |
| RASSF6 | protein_coding | turquoise |
| FAXC | protein_coding | turquoise |
| KCNJ11 | protein_coding | turquoise |
| CYP2J2 | protein_coding | turquoise |
| P3H2 | protein_coding | turquoise |
| GAD1 | protein_coding | turquoise |
| GNA14 | protein_coding | turquoise |
| RASL11B | protein_coding | turquoise |
| GALM | protein_coding | turquoise |
| XCL1 | protein_coding | turquoise |
| MIR4697HG | TEC | turquoise |
| BATF | protein_coding | turquoise |
| SLC12A2 | protein_coding | turquoise |
| SLC7A5 | protein_coding | turquoise |
| ACKR3 | protein_coding | turquoise |
| HSH2D | protein_coding | turquoise |
| ZC3H12D | protein_coding | turquoise |
| DUXAP10 | pseudogene | turquoise |
| LRRC75A | protein_coding | turquoise |
| FADS1 | protein_coding | turquoise |
| MED12L | protein_coding | turquoise |
| RETREG1 | protein_coding | turquoise |
| LRFN1 | protein_coding | turquoise |
| CA13 | protein_coding | turquoise |
| SLC4A3 | protein_coding | turquoise |
| DUXAP8 | long_non_coding | turquoise |
| TMEM51-AS1 | long_non_coding | turquoise |
| PDLIM4 | protein_coding | turquoise |
| HDGFL3 | protein_coding | turquoise |
| BCAN | protein_coding | turquoise |
| SKAP1 | protein_coding | turquoise |
| MKRN3 | protein_coding | turquoise |
| SLC44A3-AS1 | pseudogene | turquoise |
| VSTM5 | protein_coding | turquoise |
| LIF | protein_coding | turquoise |
| DISP2 | protein_coding | turquoise |
| TMEM176A | protein_coding | turquoise |
| FAM81A | protein_coding | turquoise |
| TNFRSF18 | protein_coding | turquoise |
| ARHGAP44 | protein_coding | turquoise |
| ST6GAL1 | protein_coding | turquoise |
| MGAT4A | protein_coding | turquoise |
| COBL | protein_coding | turquoise |
| SLC47A1 | protein_coding | turquoise |
| MECOM | protein_coding | turquoise |
| ZC4H2 | protein_coding | turquoise |
| PKIB | protein_coding | turquoise |
| PFN2 | protein_coding | turquoise |
| ARRB1 | protein_coding | turquoise |
| IL2RG | protein_coding | turquoise |
| KRT18 | protein_coding | turquoise |
| FAM221A | protein_coding | turquoise |
| GPR39 | protein_coding | turquoise |
| CNIH2 | protein_coding | turquoise |
| BASP1 | protein_coding | turquoise |
| CALML4 | protein_coding | turquoise |
| TMCC2 | protein_coding | turquoise |
| ADGRB1 | protein_coding | turquoise |
| UGT8 | protein_coding | turquoise |
| OR7E14P | pseudogene | turquoise |
| ZNF518B | protein_coding | turquoise |
| AL121839.2 | long_non_coding | turquoise |
| MAF | protein_coding | turquoise |
| SPIRE2 | protein_coding | turquoise |
| BMP4 | protein_coding | turquoise |
| SPART | protein_coding | turquoise |
| FBXO17 | protein_coding | turquoise |
| AL022322.1 | long_non_coding | turquoise |
| OLFM2 | protein_coding | turquoise |
| MACROD2 | protein_coding | turquoise |
| RAB37 | protein_coding | turquoise |
| SETBP1 | protein_coding | turquoise |
| LRRC37A6P | pseudogene | turquoise |
| TLDC2 | protein_coding | turquoise |
| CAV1 | protein_coding | turquoise |
| ITPKA | protein_coding | turquoise |
| GLYCTK | protein_coding | turquoise |
| SNCAIP | protein_coding | turquoise |
| BEX3 | protein_coding | turquoise |
| DUX4L50 | pseudogene | turquoise |
| C10orf55 | protein_coding | turquoise |
| PRSS12 | protein_coding | turquoise |
| LLGL2 | protein_coding | turquoise |
| ANXA9 | protein_coding | turquoise |
| NFATC2 | protein_coding | turquoise |
| ADIRF-AS1 | long_non_coding | turquoise |
| IL12RB2 | protein_coding | turquoise |
| CABLES1 | protein_coding | turquoise |
| CATSPERB | protein_coding | turquoise |
| KPNA7 | protein_coding | turquoise |
| HECW1 | protein_coding | turquoise |
| AP001972.5 | TEC | turquoise |
| PINLYP | protein_coding | turquoise |
| PODNL1 | protein_coding | turquoise |
| PDPN | protein_coding | turquoise |
| CTSS | protein_coding | turquoise |
| FAM171A1 | protein_coding | turquoise |
| SMAD6 | protein_coding | turquoise |
| FOXP4-AS1 | long_non_coding | turquoise |
| LPIN2 | protein_coding | turquoise |
| FAR2 | protein_coding | turquoise |
| SLC2A1-AS1 | long_non_coding | turquoise |
| RASEF | protein_coding | turquoise |
| CENPV | protein_coding | turquoise |
| TMEM74B | protein_coding | turquoise |
| C1QL1 | protein_coding | turquoise |
| AL117329.1 | long_non_coding | turquoise |
| PLAC4 | protein_coding | turquoise |
| TRIM36 | protein_coding | turquoise |
| ALG1L | protein_coding | turquoise |
| GALNT4 | protein_coding | turquoise |
| TSPAN15 | protein_coding | turquoise |
| AL359075.1 | pseudogene | turquoise |
| SLC37A1 | protein_coding | turquoise |
| SLFN13 | protein_coding | turquoise |
| DPF1 | protein_coding | turquoise |
| RHOU | protein_coding | turquoise |
| DTX4 | protein_coding | turquoise |
| FAM149A | protein_coding | turquoise |
| AL357033.4 | long_non_coding | turquoise |
| NCS1 | protein_coding | turquoise |
| C2orf88 | protein_coding | turquoise |
| CBX2 | protein_coding | turquoise |
| RPLP0P2 | pseudogene | turquoise |
| AC005537.1 | long_non_coding | turquoise |
| STOX2 | protein_coding | turquoise |
| AATK | protein_coding | turquoise |
| DCBLD2 | protein_coding | turquoise |
| MISP3 | protein_coding | turquoise |
| KIAA1257 | protein_coding | turquoise |
| EFCC1 | protein_coding | turquoise |
| EPHB2 | protein_coding | turquoise |
| PCDHGC3 | protein_coding | turquoise |
| BRSK2 | protein_coding | turquoise |
| MMP15 | protein_coding | turquoise |
| RAG1 | protein_coding | turquoise |
| TMEM176B | protein_coding | turquoise |
| PKP2 | protein_coding | turquoise |
| GPRIN2 | protein_coding | turquoise |
| C12orf56 | protein_coding | turquoise |
| PLTP | protein_coding | turquoise |
| ANPEP | protein_coding | turquoise |
| ACKR4 | protein_coding | turquoise |
| FZD5 | protein_coding | turquoise |
| PRICKLE1 | protein_coding | turquoise |
| EVC | protein_coding | turquoise |
| TGFBI | protein_coding | turquoise |
| TNFSF15 | protein_coding | turquoise |
| ADGRG5 | protein_coding | turquoise |
| CBARP | protein_coding | turquoise |
| ABCC6 | protein_coding | turquoise |
| TTC39A | protein_coding | turquoise |
| AC098934.1 | pseudogene | turquoise |
| SNHG26 | long_non_coding | turquoise |
| SPATA13 | protein_coding | turquoise |
| MARK1 | protein_coding | turquoise |
| MYO5C | protein_coding | turquoise |
| CHST3 | protein_coding | turquoise |
| RALGAPA2 | protein_coding | turquoise |
| PHEX | protein_coding | turquoise |
| AC002546.1 | long_non_coding | turquoise |
| AC005083.1 | long_non_coding | turquoise |
| GPRIN3 | protein_coding | turquoise |
| ADH6 | protein_coding | turquoise |
| IL1RAP | protein_coding | turquoise |
| LINC01503 | long_non_coding | turquoise |
| CSRP2 | protein_coding | turquoise |
| CELSR1 | protein_coding | turquoise |
| RPS6KL1 | protein_coding | turquoise |
| DNAH17 | protein_coding | turquoise |
| CNTLN | protein_coding | turquoise |
| ADGRE5 | protein_coding | turquoise |
| AC011379.2 | long_non_coding | turquoise |
| FRMD3 | protein_coding | turquoise |
| DZIP1 | protein_coding | turquoise |
| OSBPL7 | protein_coding | turquoise |
| KCTD15 | protein_coding | turquoise |
| FOXQ1 | protein_coding | turquoise |
| MSN | protein_coding | turquoise |
| ZNF321P | pseudogene | turquoise |
| TLR6 | protein_coding | turquoise |
| GALNT18 | protein_coding | turquoise |
| SPHK1 | protein_coding | turquoise |
| TMEM200B | protein_coding | turquoise |
| HSD17B6 | protein_coding | turquoise |
| FGD1 | protein_coding | turquoise |
| MT2A | protein_coding | turquoise |
| VWA7 | protein_coding | turquoise |
| SLC7A7 | protein_coding | turquoise |
| NRIP3 | protein_coding | turquoise |
| CREB5 | protein_coding | turquoise |
| PPP2R3A | protein_coding | turquoise |
| PARD6B | protein_coding | turquoise |
| AC004846.1 | long_non_coding | turquoise |
| MANEAL | protein_coding | turquoise |
| FOXA1 | protein_coding | turquoise |
| TMEM2 | protein_coding | turquoise |
| CAV2 | protein_coding | turquoise |
| TMEM158 | protein_coding | turquoise |
| ME3 | protein_coding | turquoise |
| SEL1L3 | protein_coding | turquoise |
| EGFR | protein_coding | turquoise |
| RAB20 | protein_coding | turquoise |
| AP001107.9 | long_non_coding | turquoise |
| LRG1 | protein_coding | turquoise |
| PLEKHH1 | protein_coding | turquoise |
| RTL8B | protein_coding | turquoise |
| KALRN | protein_coding | turquoise |
| SNTB1 | protein_coding | turquoise |
| MYO5A | protein_coding | turquoise |
| CPLX1 | protein_coding | turquoise |
| CASP10 | protein_coding | turquoise |
| GMDS | protein_coding | turquoise |
| TMEM63A | protein_coding | turquoise |
| HOXD9 | protein_coding | turquoise |
| TBC1D30 | protein_coding | turquoise |
| NUPR1 | protein_coding | turquoise |
| KIAA1644 | protein_coding | turquoise |
| LINC01679 | long_non_coding | turquoise |
| CTXN1 | protein_coding | turquoise |
| MAPK12 | protein_coding | turquoise |
| TFPI | protein_coding | turquoise |
| AP002026.1 | long_non_coding | turquoise |
| ATP8B3 | protein_coding | turquoise |
| HPDL | protein_coding | turquoise |
| GNAI1 | protein_coding | turquoise |
| HSPA2 | protein_coding | turquoise |
| PTPRJ | protein_coding | turquoise |
| CHKA | protein_coding | turquoise |
| KAZALD1 | protein_coding | turquoise |
| C1orf210 | protein_coding | turquoise |
| MTMR11 | protein_coding | turquoise |
| BCL11A | protein_coding | turquoise |
| ADGRG6 | protein_coding | turquoise |
| ERVMER34-1 | protein_coding | turquoise |
| STS | protein_coding | turquoise |
| CHST7 | protein_coding | turquoise |
| CHST11 | protein_coding | turquoise |
| PLAU | protein_coding | turquoise |
| TMEM254-AS1 | long_non_coding | turquoise |
| ACAA2 | protein_coding | turquoise |
| TMEM171 | protein_coding | turquoise |
| GPRC5C | protein_coding | turquoise |
| ZSWIM5 | protein_coding | turquoise |
| FJX1 | protein_coding | turquoise |
| LINC01410 | long_non_coding | turquoise |
| KRT19 | protein_coding | turquoise |
| HEY1 | protein_coding | turquoise |
| ARHGAP42 | protein_coding | turquoise |
| GPR161 | protein_coding | turquoise |
| ARSJ | protein_coding | turquoise |
| MYH10 | protein_coding | turquoise |
| PPP1R3G | protein_coding | turquoise |
| CAMK1D | protein_coding | turquoise |
| PLXNB3 | protein_coding | turquoise |
| CHST2 | protein_coding | turquoise |
| MLLT11 | protein_coding | turquoise |
| MST1R | protein_coding | turquoise |
| ASS1 | protein_coding | turquoise |
| FAM174B | protein_coding | turquoise |
| LIPG | protein_coding | turquoise |
| NSUN7 | protein_coding | turquoise |
| PIK3AP1 | protein_coding | turquoise |
| CD55 | protein_coding | turquoise |
| RALGPS1 | protein_coding | turquoise |
| HNMT | protein_coding | turquoise |
| RPH3AL | protein_coding | turquoise |
| DNAJB5 | protein_coding | turquoise |
| PRNP | protein_coding | turquoise |
| NETO2 | protein_coding | turquoise |
| WNT2B | protein_coding | turquoise |
| EPS8 | protein_coding | turquoise |
| ASL | protein_coding | turquoise |
| DGCR6 | protein_coding | turquoise |
| TUSC1 | protein_coding | turquoise |
| NXN | protein_coding | turquoise |
| ELOVL5 | protein_coding | turquoise |
| FMN1 | protein_coding | turquoise |
| HSD11B2 | protein_coding | turquoise |
| LGALS9 | protein_coding | turquoise |
| ERBB3 | protein_coding | turquoise |
| TMEM38A | protein_coding | turquoise |
| PLBD1 | protein_coding | turquoise |
| C2 | protein_coding | turquoise |
| AL035252.3 | long_non_coding | turquoise |
| AC093110.1 | long_non_coding | turquoise |
| ARHGAP26 | protein_coding | turquoise |
| FAM107B | protein_coding | turquoise |
| NPDC1 | protein_coding | turquoise |
| NUDT16P1 | pseudogene | turquoise |
| FOXD2 | protein_coding | turquoise |
| GPR68 | protein_coding | turquoise |
| POU5F1 | protein_coding | turquoise |
| PIP5KL1 | protein_coding | turquoise |
| CLTCL1 | protein_coding | turquoise |
| TNFRSF1B | protein_coding | turquoise |
| ACE | protein_coding | turquoise |
| DGAT2 | protein_coding | turquoise |
| MKRN2OS | protein_coding | turquoise |
| PARD3B | protein_coding | turquoise |
| HSD17B11 | protein_coding | turquoise |
| APCDD1 | protein_coding | turquoise |
| CADPS2 | protein_coding | turquoise |
| FN3K | protein_coding | turquoise |
| PLLP | protein_coding | turquoise |
| NOL4L | protein_coding | turquoise |
| SEZ6L2 | protein_coding | turquoise |
| CDH13 | protein_coding | turquoise |
| NLRP1 | protein_coding | turquoise |
| AC013652.1 | long_non_coding | turquoise |
| KLF7 | protein_coding | turquoise |
| LGR4 | protein_coding | turquoise |
| CRMP1 | protein_coding | turquoise |
| CRIP1 | protein_coding | turquoise |
| PNKD | protein_coding | turquoise |
| DAPK2 | protein_coding | turquoise |
| FAM109A | protein_coding | turquoise |
| HOXD8 | protein_coding | turquoise |
| TMEM132A | protein_coding | turquoise |
| FEZ1 | protein_coding | turquoise |
| 3-Mar | protein_coding | turquoise |
| AXIN2 | protein_coding | turquoise |
| FGF1 | protein_coding | turquoise |
| AC108058.1 | long_non_coding | turquoise |
| FLNA | protein_coding | turquoise |
| KRBA1 | protein_coding | turquoise |
| TGFB1 | protein_coding | turquoise |
| KIF13B | protein_coding | turquoise |
| ARHGEF19 | protein_coding | turquoise |
| ISG20 | protein_coding | turquoise |
| LZTS3 | protein_coding | turquoise |
| RPL39L | protein_coding | turquoise |
| LARP6 | protein_coding | turquoise |
| TMC7 | protein_coding | turquoise |
| SLC52A3 | protein_coding | turquoise |
| MAGIX | protein_coding | turquoise |
| GPT | protein_coding | turquoise |
| RIMKLB | protein_coding | turquoise |
| GALNT12 | protein_coding | turquoise |
| CMYA5 | protein_coding | turquoise |
| ZNF287 | protein_coding | turquoise |
| AK7 | protein_coding | turquoise |
| LINC00342 | long_non_coding | turquoise |
| PGPEP1 | protein_coding | turquoise |
| OBSL1 | protein_coding | turquoise |
| KIAA1161 | protein_coding | turquoise |
| RNF125 | protein_coding | turquoise |
| SDSL | protein_coding | turquoise |
| SLC46A3 | protein_coding | turquoise |
| TRPM2-AS | long_non_coding | turquoise |
| EEPD1 | protein_coding | turquoise |
| PARD6A | protein_coding | turquoise |
| AP003119.3 | long_non_coding | turquoise |
| GNB4 | protein_coding | turquoise |
| TC2N | protein_coding | turquoise |
| TMEM133 | protein_coding | turquoise |
| LBX2-AS1 | long_non_coding | turquoise |
| CTH | protein_coding | turquoise |
| CASC15 | long_non_coding | turquoise |
| NOTCH3 | protein_coding | turquoise |
| FZD6 | protein_coding | turquoise |
| KTN1-AS1 | long_non_coding | turquoise |
| ARL10 | protein_coding | turquoise |
| GPSM1 | protein_coding | turquoise |
| AL024508.2 | long_non_coding | turquoise |
| NANOS1 | protein_coding | turquoise |
| TNFRSF10D | protein_coding | turquoise |
| HES6 | protein_coding | turquoise |
| KCNQ1OT1 | long_non_coding | turquoise |
| SLC1A4 | protein_coding | turquoise |
| AP000769.1 | pseudogene | turquoise |
| ANXA3 | protein_coding | turquoise |
| ARL4C | protein_coding | turquoise |
| C1orf132 | long_non_coding | turquoise |
| FAM105A | protein_coding | turquoise |
| SPATA6L | protein_coding | turquoise |
| C2orf15 | protein_coding | turquoise |
| FAM234B | protein_coding | turquoise |
| TLN2 | protein_coding | turquoise |
| ZNF503-AS2 | long_non_coding | turquoise |
| MAP2K6 | protein_coding | turquoise |
| CCND1 | protein_coding | turquoise |
| AC073957.3 | long_non_coding | turquoise |
| LY75 | protein_coding | turquoise |
| NLGN2 | protein_coding | turquoise |
| METRN | protein_coding | turquoise |
| VWA5A | protein_coding | turquoise |
| SH3KBP1 | protein_coding | turquoise |
| SLC12A7 | protein_coding | turquoise |
| WWC1 | protein_coding | turquoise |
| LFNG | protein_coding | turquoise |
| SRGAP3 | protein_coding | turquoise |
| IRAK2 | protein_coding | turquoise |
| ZMYND15 | protein_coding | turquoise |
| SCPEP1 | protein_coding | turquoise |
| PNPLA7 | protein_coding | turquoise |
| ZNF608 | protein_coding | turquoise |
| KRT8P33 | pseudogene | turquoise |
| ACRBP | protein_coding | turquoise |
| TCTEX1D2 | protein_coding | turquoise |
| JAG2 | protein_coding | turquoise |
| FHIT | protein_coding | turquoise |
| STAMBPL1 | protein_coding | turquoise |
| ZNF512B | protein_coding | turquoise |
| HS3ST3B1 | protein_coding | turquoise |
| PLCE1 | protein_coding | turquoise |
| C11orf45 | protein_coding | turquoise |
| TMC4 | protein_coding | turquoise |
| BFSP1 | protein_coding | turquoise |
| PLOD2 | protein_coding | turquoise |
| NOXA1 | protein_coding | turquoise |
| HLA-J | pseudogene | turquoise |
| RAB31 | protein_coding | turquoise |
| ZNF137P | pseudogene | turquoise |
| KIF21B | protein_coding | turquoise |
| ELL2 | protein_coding | turquoise |
| ATP11A | protein_coding | turquoise |
| ANO7 | protein_coding | turquoise |
| MARVELD1 | protein_coding | turquoise |
| DAGLA | protein_coding | turquoise |
| ATP1B1 | protein_coding | turquoise |
| KIF3C | protein_coding | turquoise |
| CAVIN1 | protein_coding | turquoise |
| RAB11FIP4 | protein_coding | turquoise |
| ANXA4 | protein_coding | turquoise |
| ADCY6 | protein_coding | turquoise |
| CPT1C | protein_coding | turquoise |
| RIPK3 | protein_coding | turquoise |
| SVIP | protein_coding | turquoise |
| C21orf91 | protein_coding | turquoise |
| PODXL | protein_coding | turquoise |
| PGF | protein_coding | turquoise |
| GALE | protein_coding | turquoise |
| TNFSF13 | protein_coding | turquoise |
| SIAE | protein_coding | turquoise |
| LDHB | protein_coding | turquoise |
| DSE | protein_coding | turquoise |
| RBM47 | protein_coding | turquoise |
| MCF2L | protein_coding | turquoise |
| CDHR3 | protein_coding | turquoise |
| BIK | protein_coding | turquoise |
| ZNF559 | protein_coding | turquoise |
| ZSCAN12P1 | pseudogene | turquoise |
| BIN1 | protein_coding | turquoise |
| CASC10 | protein_coding | turquoise |
| MAGI1 | protein_coding | turquoise |
| NFE2L3 | protein_coding | turquoise |
| C9orf84 | protein_coding | turquoise |
| TSPAN13 | protein_coding | turquoise |
| EHD2 | protein_coding | turquoise |
| SOX9 | protein_coding | turquoise |
| HACD1 | protein_coding | turquoise |
| ABHD2 | protein_coding | turquoise |
| BCL6 | protein_coding | turquoise |
| TPK1 | protein_coding | turquoise |
| AC084033.3 | long_non_coding | turquoise |
| C2orf82 | protein_coding | turquoise |
| GSEC | long_non_coding | turquoise |
| PFKFB2 | protein_coding | turquoise |
| CMTM7 | protein_coding | turquoise |
| B3GNT4 | protein_coding | turquoise |
| GAL3ST4 | protein_coding | turquoise |
| LINC01094 | long_non_coding | turquoise |
| SIRPA | protein_coding | turquoise |
| SPRY2 | protein_coding | turquoise |
| AL139393.2 | long_non_coding | turquoise |
| IGF1R | protein_coding | turquoise |
| FAM46A | protein_coding | turquoise |
| FUCA1 | protein_coding | turquoise |
| HOXA4 | protein_coding | turquoise |
| TCF7L1 | protein_coding | turquoise |
| LGALS3 | protein_coding | turquoise |
| SLC45A3 | protein_coding | turquoise |
| RNASET2 | protein_coding | turquoise |
| AGAP2-AS1 | long_non_coding | turquoise |
| IL7 | protein_coding | turquoise |
| TNFRSF14 | protein_coding | turquoise |
| LINC01123 | long_non_coding | turquoise |
| SIGIRR | protein_coding | turquoise |
| EPB41L4B | protein_coding | turquoise |
| RFK | protein_coding | turquoise |
| ZNF91 | protein_coding | turquoise |
| ATF7IP2 | protein_coding | turquoise |
| AUTS2 | protein_coding | turquoise |
| PGGHG | protein_coding | turquoise |
| TSPAN3 | protein_coding | turquoise |
| PMAIP1 | protein_coding | turquoise |
| SPTBN1 | protein_coding | turquoise |
| CPNE8 | protein_coding | turquoise |
| AL591895.1 | long_non_coding | turquoise |
| PRKCA | protein_coding | turquoise |
| C1GALT1 | protein_coding | turquoise |
| CEP19 | protein_coding | turquoise |
| AL390719.2 | long_non_coding | turquoise |
| RHOF | protein_coding | turquoise |
| AP1S3 | protein_coding | turquoise |
| ACP6 | protein_coding | turquoise |
| GPM6B | protein_coding | turquoise |
| ZNF701 | protein_coding | turquoise |
| FBXL19-AS1 | long_non_coding | turquoise |
| COTL1 | protein_coding | turquoise |
| MCU | protein_coding | turquoise |
| LINC00926 | long_non_coding | turquoise |
| MDFIC | protein_coding | turquoise |
| ZDHHC23 | protein_coding | turquoise |
| EIF3EP1 | pseudogene | turquoise |
| AL121832.3 | long_non_coding | turquoise |
| PAPSS2 | protein_coding | turquoise |
| ABTB2 | protein_coding | turquoise |
| MXRA7 | protein_coding | turquoise |
| DOK4 | protein_coding | turquoise |
| GCC2 | protein_coding | turquoise |
| OBSCN | protein_coding | turquoise |
| AC022364.1 | long_non_coding | turquoise |
| AC009948.1 | long_non_coding | turquoise |
| GCNA | protein_coding | turquoise |
| HS6ST1 | protein_coding | turquoise |
| SNAPC1 | protein_coding | turquoise |
| KIAA1211L | protein_coding | turquoise |
| CMTM8 | protein_coding | turquoise |
| NCOA7 | protein_coding | turquoise |
| AL590617.2 | long_non_coding | turquoise |
| AJUBA | protein_coding | turquoise |
| ZNF860 | protein_coding | turquoise |
| CTSH | protein_coding | turquoise |
| C1orf115 | protein_coding | turquoise |
| SLC25A20 | protein_coding | turquoise |
| MTND6P4 | pseudogene | turquoise |
| CYBA | protein_coding | turquoise |
| ANKRD36C | protein_coding | turquoise |
| DST | protein_coding | turquoise |
| ARHGEF10L | protein_coding | turquoise |
| AC015883.1 | TEC | turquoise |
| TMEM144 | protein_coding | turquoise |
| SCD5 | protein_coding | turquoise |
| WDR91 | protein_coding | turquoise |
| PSTPIP2 | protein_coding | turquoise |
| TMEM237 | protein_coding | turquoise |
| AL590627.1 | pseudogene | turquoise |
| ST3GAL3 | protein_coding | turquoise |
| AL391988.1 | long_non_coding | turquoise |
| TTC7B | protein_coding | turquoise |
| MIR17HG | long_non_coding | turquoise |
| CDR2L | protein_coding | turquoise |
| FCGRT | protein_coding | turquoise |
| ZNF774 | protein_coding | turquoise |
| NEK3 | protein_coding | turquoise |
| DGKD | protein_coding | turquoise |
| ACY1 | protein_coding | turquoise |
| PCSK6 | protein_coding | turquoise |
| LINC01106 | long_non_coding | turquoise |
| ABHD11 | protein_coding | turquoise |
| PIK3C2B | protein_coding | turquoise |
| KDELR3 | protein_coding | turquoise |
| WASF1 | protein_coding | turquoise |
| PANK1 | protein_coding | turquoise |
| GNE | protein_coding | turquoise |
| TPBG | protein_coding | turquoise |
| SSBP2 | protein_coding | turquoise |
| PTK7 | protein_coding | turquoise |
| TP53I11 | protein_coding | turquoise |
| ARHGAP18 | protein_coding | turquoise |
| AL138756.1 | long_non_coding | turquoise |
| SLC38A2 | protein_coding | turquoise |
| NAT1 | protein_coding | turquoise |
| SLC26A1 | protein_coding | turquoise |
| KIAA1024 | protein_coding | turquoise |
| ZNF816 | protein_coding | turquoise |
| AC128688.2 | long_non_coding | turquoise |
| STARD10 | protein_coding | turquoise |
| PBX4 | protein_coding | turquoise |
| GRK5 | protein_coding | turquoise |
| ASPHD2 | protein_coding | turquoise |
| CDC42EP2 | protein_coding | turquoise |
| NEK5 | protein_coding | turquoise |
| ID2 | protein_coding | turquoise |
| GAB2 | protein_coding | turquoise |
| MSX2 | protein_coding | turquoise |
| HENMT1 | protein_coding | turquoise |
| GRID2IP | protein_coding | turquoise |
| DNAH1 | protein_coding | turquoise |
| VSIG10 | protein_coding | turquoise |
| C2CD4D | protein_coding | turquoise |
| DUSP8 | protein_coding | turquoise |
| MTCO1P2 | pseudogene | turquoise |
| AC010186.2 | pseudogene | turquoise |
| GFPT1 | protein_coding | turquoise |
| TGFBR2 | protein_coding | turquoise |
| DHRS11 | protein_coding | turquoise |
| 2-Mar | protein_coding | turquoise |
| PBLD | protein_coding | turquoise |
| TPRN | protein_coding | turquoise |
| GDPD5 | protein_coding | turquoise |
| NCEH1 | protein_coding | turquoise |
| CYP4V2 | protein_coding | turquoise |
| CXXC5 | protein_coding | turquoise |
| ZFPM1 | protein_coding | turquoise |
| FAM126A | protein_coding | turquoise |
| CLDN15 | protein_coding | turquoise |
| EXOC3-AS1 | long_non_coding | turquoise |
| MAPK11 | protein_coding | turquoise |
| ZSCAN16 | protein_coding | turquoise |
| SPSB1 | protein_coding | turquoise |
| TP53TG1 | long_non_coding | turquoise |
| AL158206.1 | long_non_coding | turquoise |
| SLC45A4 | protein_coding | turquoise |
| CHPT1 | protein_coding | turquoise |
| THAP7-AS1 | long_non_coding | turquoise |
| SLC39A6 | protein_coding | turquoise |
| TMTC3 | protein_coding | turquoise |
| SLC16A5 | protein_coding | turquoise |
| XYLB | protein_coding | turquoise |
| NPL | protein_coding | turquoise |
| PTGFRN | protein_coding | turquoise |
| INPP5J | protein_coding | turquoise |
| AL359643.3 | long_non_coding | turquoise |
| RAP1GAP2 | protein_coding | turquoise |
| DBN1 | protein_coding | turquoise |
| FAM213B | protein_coding | turquoise |
| RRN3P1 | pseudogene | turquoise |
| CDH24 | protein_coding | turquoise |
| SLC2A13 | protein_coding | turquoise |
| MTSS1L | protein_coding | turquoise |
| BLVRA | protein_coding | turquoise |
| TINAGL1 | protein_coding | turquoise |
| ZNF792 | protein_coding | turquoise |
| PLXNA1 | protein_coding | turquoise |
| ZNF655 | protein_coding | turquoise |
| CRYL1 | protein_coding | turquoise |
| ARHGAP12 | protein_coding | turquoise |
| PRRG1 | protein_coding | turquoise |
| MBOAT1 | protein_coding | turquoise |
| KIF9 | protein_coding | turquoise |
| FADD | protein_coding | turquoise |
| CDIP1 | protein_coding | turquoise |
| P4HTM | protein_coding | turquoise |
| MTCO1P40 | pseudogene | turquoise |
| SMPDL3A | protein_coding | turquoise |
| TMEM17 | protein_coding | turquoise |
| EVL | protein_coding | turquoise |
| C18orf54 | protein_coding | turquoise |
| TANC2 | protein_coding | turquoise |
| DNAJB4 | protein_coding | turquoise |
| MYO6 | protein_coding | turquoise |
| CA11 | protein_coding | turquoise |
| SLC29A2 | protein_coding | turquoise |
| SLC3A2 | protein_coding | turquoise |
| ACBD4 | protein_coding | turquoise |
| PALLD | protein_coding | turquoise |
| INSR | protein_coding | turquoise |
| TEAD2 | protein_coding | turquoise |
| CTSC | protein_coding | turquoise |
| DOCK5 | protein_coding | turquoise |
| CACNA2D4 | protein_coding | turquoise |
| DUSP14 | protein_coding | turquoise |
| OSBPL10 | protein_coding | turquoise |
| FOXD2-AS1 | long_non_coding | turquoise |
| VDAC1P8 | pseudogene | turquoise |
| AC055811.4 | TEC | turquoise |
| ERBB2 | protein_coding | turquoise |
| PHYH | protein_coding | turquoise |
| AL359220.1 | long_non_coding | turquoise |
| NUDT7 | protein_coding | turquoise |
| ZFYVE28 | protein_coding | turquoise |
| SLC35D2 | protein_coding | turquoise |
| GCHFR | protein_coding | turquoise |
| SNHG22 | long_non_coding | turquoise |
| TMEM62 | protein_coding | turquoise |
| DGAT1 | protein_coding | turquoise |
| GK | protein_coding | turquoise |
| KATNAL1 | protein_coding | turquoise |
| SOGA1 | protein_coding | turquoise |
| SMCO4 | protein_coding | turquoise |
| PIK3CD | protein_coding | turquoise |
| CDCA4 | protein_coding | turquoise |
| SFMBT1 | protein_coding | turquoise |
| PDZD8 | protein_coding | turquoise |
| KIAA1614 | protein_coding | turquoise |
| BACE2 | protein_coding | turquoise |
| OVGP1 | protein_coding | turquoise |
| MGAT5 | protein_coding | turquoise |
| JARID2 | protein_coding | turquoise |
| AHCYL2 | protein_coding | turquoise |
| DUBR | long_non_coding | turquoise |
| GSTO2 | protein_coding | turquoise |
| ACTL10 | protein_coding | turquoise |
| MARCKSL1 | protein_coding | turquoise |
| SLC18B1 | protein_coding | turquoise |
| CYB561D2 | protein_coding | turquoise |
| NR3C1 | protein_coding | turquoise |
| FGD4 | protein_coding | turquoise |
| AC019205.2 | long_non_coding | turquoise |
| AL353622.1 | long_non_coding | turquoise |
| AQP11 | protein_coding | turquoise |
| SCML1 | protein_coding | turquoise |
| SUSD1 | protein_coding | turquoise |
| UNC13B | protein_coding | turquoise |
| SPRY4 | protein_coding | turquoise |
| TPST1 | protein_coding | turquoise |
| LYN | protein_coding | turquoise |
| SLC17A5 | protein_coding | turquoise |
| CLCN5 | protein_coding | turquoise |
| C3orf52 | protein_coding | turquoise |
| NME4 | protein_coding | turquoise |
| TCEA2 | protein_coding | turquoise |
| CACFD1 | protein_coding | turquoise |
| MTCO1P53 | pseudogene | turquoise |
