# Supplementary material for: Integrated bioinformatics analysis to decipher molecular mechanism of compound Kushen injection for esophageal cancer by combining WGCNA with network pharmacology
Source: Sci Rep. 2020 Jul 29;10:12745. doi: 10.1038/s41598-020-69708-2 (PMC7391752; doi:10.1038/s41598-020-69708-2)
Supplement: Supplementary file 2 — Supplementary file2 [file 41598_2020_69708_MOESM2_ESM.docx]

**Integrated Bioinformatics Analysis to Decipher Molecular Mechanism of Compound Kushen Injection for Esophageal Cancer by Combining WGCNA with Network Pharmacology**

**Wei Zhou^1^, Jiarui Wu^1^*****, Jingyuan Zhang^1^, Xinkui Liu^1^, Siyu Guo^1^, ShanShan Jia^1^, Xiaomeng Zhang^1^, Yingli Zhu^1^, Miaomiao Wang^1^**

1Beijing University of Chinese Medicine, Beijing, 100102, China

* Corresponding email: exogamy@163.com

| nodeName | altName | nodeAttr[nodesPresent, ] |
| --- | --- | --- |
| ADH7 | protein_coding | blue |
| SPRR3 | protein_coding | blue |
| IVL | protein_coding | blue |
| KRTDAP | protein_coding | blue |
| KRT6C | protein_coding | blue |
| SBSN | protein_coding | blue |
| SPRR2E | protein_coding | blue |
| KRT13 | protein_coding | blue |
| KRT4 | protein_coding | blue |
| KRT14 | protein_coding | blue |
| TMPRSS11A | protein_coding | blue |
| A2ML1 | protein_coding | blue |
| TMPRSS11D | protein_coding | blue |
| S100A7A | protein_coding | blue |
| SPRR2A | protein_coding | blue |
| FOXE1 | protein_coding | blue |
| KRT75 | protein_coding | blue |
| SPRR1A | protein_coding | blue |
| CALML5 | protein_coding | blue |
| S100A7 | protein_coding | blue |
| CAPNS2 | protein_coding | blue |
| CRCT1 | protein_coding | blue |
| IRX4 | protein_coding | blue |
| SPRR2D | protein_coding | blue |
| C10orf99 | protein_coding | blue |
| RHCG | protein_coding | blue |
| SPRR1B | protein_coding | blue |
| IL36G | protein_coding | blue |
| TMPRSS11E | protein_coding | blue |
| SERPINB13 | protein_coding | blue |
| IL36RN | protein_coding | blue |
| LY6D | protein_coding | blue |
| LGALS7 | protein_coding | blue |
| KLK5 | protein_coding | blue |
| GJB6 | protein_coding | blue |
| FAM83C | protein_coding | blue |
| KRT16 | protein_coding | blue |
| LGALS7B | protein_coding | blue |
| TENM2 | protein_coding | blue |
| GBP6 | protein_coding | blue |
| NEFL | protein_coding | blue |
| SLC6A11 | protein_coding | blue |
| S100A8 | protein_coding | blue |
| SERPINB3 | protein_coding | blue |
| TGM3 | protein_coding | blue |
| CLCA4 | protein_coding | blue |
| KRT16P6 | pseudogene | blue |
| KRT6B | protein_coding | blue |
| DSG1 | protein_coding | blue |
| BNC1 | protein_coding | blue |
| SERPINB4 | protein_coding | blue |
| WFDC5 | protein_coding | blue |
| RNASE7 | protein_coding | blue |
| KRT78 | protein_coding | blue |
| PGLYRP3 | protein_coding | blue |
| NCCRP1 | protein_coding | blue |
| KLK13 | protein_coding | blue |
| TGM1 | protein_coding | blue |
| FOXN1 | protein_coding | blue |
| S100A9 | protein_coding | blue |
| CALML3 | protein_coding | blue |
| DAPL1 | protein_coding | blue |
| DMRTA2 | protein_coding | blue |
| KRT6A | protein_coding | blue |
| CSTA | protein_coding | blue |
| SCEL | protein_coding | blue |
| CDHR1 | protein_coding | blue |
| TP63 | protein_coding | blue |
| CPA4 | protein_coding | blue |
| PKP1 | protein_coding | blue |
| FAT2 | protein_coding | blue |
| LYPD2 | protein_coding | blue |
| CERS3 | protein_coding | blue |
| IGFL1 | protein_coding | blue |
| ZNF812P | pseudogene | blue |
| GSDMC | protein_coding | blue |
| PTHLH | protein_coding | blue |
| DSG3 | protein_coding | blue |
| DSC3 | protein_coding | blue |
| USH1G | protein_coding | blue |
| S100A2 | protein_coding | blue |
| NIPAL4 | protein_coding | blue |
| SLC39A2 | protein_coding | blue |
| SERPINB2 | protein_coding | blue |
| ZNF750 | protein_coding | blue |
| TP53AIP1 | protein_coding | blue |
| CNFN | protein_coding | blue |
| CLCA2 | protein_coding | blue |
| KRT5 | protein_coding | blue |
| LYPD3 | protein_coding | blue |
| SOX2 | protein_coding | blue |
| PLA2G4E | protein_coding | blue |
| TMEM40 | protein_coding | blue |
| MUC15 | protein_coding | blue |
| FGFBP1 | protein_coding | blue |
| KLK12 | protein_coding | blue |
| SOX15 | protein_coding | blue |
| TINCR | protein_coding | blue |
| MIR205HG | long_non_coding | blue |
| AKR1B15 | protein_coding | blue |
| HEPHL1 | protein_coding | blue |
| KRT17 | protein_coding | blue |
| GABRQ | protein_coding | blue |
| SCNN1B | protein_coding | blue |
| MAL | protein_coding | blue |
| CALML3-AS1 | long_non_coding | blue |
| LY6G6C | protein_coding | blue |
| PPP1R9A | protein_coding | blue |
| SOX21 | protein_coding | blue |
| S1PR5 | protein_coding | blue |
| PTPRZ1 | protein_coding | blue |
| PPP2R2C | protein_coding | blue |
| PGLYRP4 | protein_coding | blue |
| EPHX3 | protein_coding | blue |
| PI3 | protein_coding | blue |
| FLG | protein_coding | blue |
| GRHL3 | protein_coding | blue |
| KRT15 | protein_coding | blue |
| S100A12 | protein_coding | blue |
| ANXA8 | protein_coding | blue |
| GPR87 | protein_coding | blue |
| PADI1 | protein_coding | blue |
| IGFL2-AS1 | long_non_coding | blue |
| LPAR3 | protein_coding | blue |
| ANXA8L1 | protein_coding | blue |
| FAM83A | protein_coding | blue |
| CAPN14 | protein_coding | blue |
| COL17A1 | protein_coding | blue |
| SOX21-AS1 | long_non_coding | blue |
| SCNN1G | protein_coding | blue |
| FZD10 | protein_coding | blue |
| CST6 | protein_coding | blue |
| IL20RB | protein_coding | blue |
| RGS20 | protein_coding | blue |
| SUSD4 | protein_coding | blue |
| IL1A | protein_coding | blue |
| CALB2 | protein_coding | blue |
| FABP5 | protein_coding | blue |
| GABRE | protein_coding | blue |
| AL161431.1 | long_non_coding | blue |
| SPINK5 | protein_coding | blue |
| TM4SF19 | protein_coding | blue |
| AL033384.1 | long_non_coding | blue |
| LINC01605 | long_non_coding | blue |
| ALDH3B2 | protein_coding | blue |
| DLX3 | protein_coding | blue |
| BNIPL | protein_coding | blue |
| AQP3 | protein_coding | blue |
| TMEM63C | protein_coding | blue |
| KLK7 | protein_coding | blue |
| SERPINB7 | protein_coding | blue |
| P2RY1 | protein_coding | blue |
| ZBED2 | protein_coding | blue |
| RGL3 | protein_coding | blue |
| WNT7B | protein_coding | blue |
| XG | protein_coding | blue |
| FAM83A-AS1 | long_non_coding | blue |
| SPAG17 | protein_coding | blue |
| PGBD5 | protein_coding | blue |
| RAET1L | protein_coding | blue |
| PADI3 | protein_coding | blue |
| ZNF365 | protein_coding | blue |
| HES2 | protein_coding | blue |
| HCAR3 | protein_coding | blue |
| SLC10A6 | protein_coding | blue |
| KLK8 | protein_coding | blue |
| GJA3 | protein_coding | blue |
| HCAR2 | protein_coding | blue |
| ELOVL4 | protein_coding | blue |
| ARHGAP40 | protein_coding | blue |
| DHRS9 | protein_coding | blue |
| ARL4D | protein_coding | blue |
| ABCA13 | protein_coding | blue |
| AC245041.1 | long_non_coding | blue |
| HAS3 | protein_coding | blue |
| GJB2 | protein_coding | blue |
| KLK11 | protein_coding | blue |
| RAB38 | protein_coding | blue |
| APOBEC3A | protein_coding | blue |
| SDK2 | protein_coding | blue |
| KLK6 | protein_coding | blue |
| FABP5P7 | pseudogene | blue |
| KLC3 | protein_coding | blue |
| CXCL14 | protein_coding | blue |
| GJB5 | protein_coding | blue |
| HSPB8 | protein_coding | blue |
| RGMA | protein_coding | blue |
| AC008556.1 | long_non_coding | blue |
| CRABP2 | protein_coding | blue |
| NDRG4 | protein_coding | blue |
| EFS | protein_coding | blue |
| SLC16A9 | protein_coding | blue |
| TPRXL | pseudogene | blue |
| HS3ST3A1 | protein_coding | blue |
| ASPG | protein_coding | blue |
| PPP1R14C | protein_coding | blue |
| ALOXE3 | protein_coding | blue |
| PLCH2 | protein_coding | blue |
| ALOX15B | protein_coding | blue |
| ADRB2 | protein_coding | blue |
| DENND2C | protein_coding | blue |
| WFDC21P | long_non_coding | blue |
| KLK10 | protein_coding | blue |
| CA12 | protein_coding | blue |
| LYNX1 | protein_coding | blue |
| EDN2 | protein_coding | blue |
| NRG1 | protein_coding | blue |
| PALM3 | protein_coding | blue |
| BTBD11 | protein_coding | blue |
| ARHGEF4 | protein_coding | blue |
| IRX3 | protein_coding | blue |
| LGALS9B | protein_coding | blue |
| RAET1G | protein_coding | blue |
| PARD6G | protein_coding | blue |
| ALOX12B | protein_coding | blue |
| AC245041.2 | long_non_coding | blue |
| SFN | protein_coding | blue |
| PAX9 | protein_coding | blue |
| TENM4 | protein_coding | blue |
| TRIM29 | protein_coding | blue |
| CYSRT1 | protein_coding | blue |
| RAET1E | protein_coding | blue |
| AC011337.1 | long_non_coding | blue |
| ANXA1 | protein_coding | blue |
| PRSS27 | protein_coding | blue |
| RHOV | protein_coding | blue |
| ABCA12 | protein_coding | blue |
| VSIG10L | protein_coding | blue |
| SRRM3 | protein_coding | blue |
| RAB7B | protein_coding | blue |
| TPRG1 | protein_coding | blue |
| PTPN13 | protein_coding | blue |
| DUOXA1 | protein_coding | blue |
| NDUFA4L2 | protein_coding | blue |
| PNMA2 | protein_coding | blue |
| UPK3B | protein_coding | blue |
| FAM46B | protein_coding | blue |
| CRYAB | protein_coding | blue |
| SULT2B1 | protein_coding | blue |
| LGALS9C | protein_coding | blue |
| CLIC3 | protein_coding | blue |
| HGNC:18790 | protein_coding | blue |
| ANKRD35 | protein_coding | blue |
| VSNL1 | protein_coding | blue |
| IL1RN | protein_coding | blue |
| LTB4R | protein_coding | blue |
| KLF8 | protein_coding | blue |
| ST6GALNAC2 | protein_coding | blue |
| PYGL | protein_coding | blue |
| 5-Sep | protein_coding | blue |
| WNT10A | protein_coding | blue |
| CD109 | protein_coding | blue |
| GPC1 | protein_coding | blue |
| DSC2 | protein_coding | blue |
| FRMD6 | protein_coding | blue |
| ENTPD3 | protein_coding | blue |
| SH3PXD2A-AS1 | long_non_coding | blue |
| DNASE1L3 | protein_coding | blue |
| CDH26 | protein_coding | blue |
| AHNAK2 | protein_coding | blue |
| GNA15 | protein_coding | blue |
| RNF217 | protein_coding | blue |
| HSPB1 | protein_coding | blue |
| AC018629.1 | TEC | blue |
| PRODH | protein_coding | blue |
| TMEM154 | protein_coding | blue |
| TNNT2 | protein_coding | blue |
| GOLGA7B | protein_coding | blue |
| NECTIN1 | protein_coding | blue |
| ZNF385A | protein_coding | blue |
| SH2D5 | protein_coding | blue |
| MCC | protein_coding | blue |
| TFAP2C | protein_coding | blue |
| MTSS1 | protein_coding | blue |
| GRB14 | protein_coding | blue |
| TMPRSS13 | protein_coding | blue |
| LRRC4 | protein_coding | blue |
| CHRNB4 | protein_coding | blue |
| HSPB1P1 | pseudogene | blue |
| PPP1R3C | protein_coding | blue |
| CXCR2 | protein_coding | blue |
| TMEM79 | protein_coding | blue |
| THBD | protein_coding | blue |
| ZNF185 | protein_coding | blue |
| PERP | protein_coding | blue |
| ADGRF4 | protein_coding | blue |
| TIAM1 | protein_coding | blue |
| SYNGR1 | protein_coding | blue |
| VAV3 | protein_coding | blue |
| TRIM7 | protein_coding | blue |
| CACNA2D3 | protein_coding | blue |
| AATBC | long_non_coding | blue |
| FAM212A | protein_coding | blue |
| DSP | protein_coding | blue |
| BBOX1 | protein_coding | blue |
| HOPX | protein_coding | blue |
| RDH16 | protein_coding | blue |
| DMKN | protein_coding | blue |
| GJB4 | protein_coding | blue |
| PPL | protein_coding | blue |
| CELSR2 | protein_coding | blue |
| AP002761.4 | long_non_coding | blue |
| SERPINB5 | protein_coding | blue |
| NOD2 | protein_coding | blue |
| HSPB1P2 | pseudogene | blue |
| IGFBP6 | protein_coding | blue |
| FAM131C | protein_coding | blue |
| HAAO | protein_coding | blue |
| A4GALT | protein_coding | blue |
| TSLP | protein_coding | blue |
| S100A14 | protein_coding | blue |
| STON2 | protein_coding | blue |
| TUBB6 | protein_coding | blue |
| NDRG1 | protein_coding | blue |
| ACAN | protein_coding | blue |
| AL445933.1 | pseudogene | blue |
| SLC37A2 | protein_coding | blue |
| BICD2 | protein_coding | blue |
| CDK5R1 | protein_coding | blue |
| VEPH1 | protein_coding | blue |
| TPD52L1 | protein_coding | blue |
| SESN3 | protein_coding | blue |
| NCKAP5 | protein_coding | blue |
| P2RY2 | protein_coding | blue |
| MAFB | protein_coding | blue |
| LTB4R2 | protein_coding | blue |
| FGFR3 | protein_coding | blue |
| SLC2A1 | protein_coding | blue |
| TFAP2A | protein_coding | blue |
| AL139385.1 | long_non_coding | blue |
| FKBP10 | protein_coding | blue |
| SLPI | protein_coding | blue |
| DUOX1 | protein_coding | blue |
| GLTP | protein_coding | blue |
| SLC9A9 | protein_coding | blue |
| NKPD1 | protein_coding | blue |
| NECTIN4 | protein_coding | blue |
| AL109976.1 | long_non_coding | blue |
| CLIP4 | protein_coding | blue |
| TNS4 | protein_coding | blue |
| ENPP1 | protein_coding | blue |
| HOXC13 | protein_coding | blue |
| OXER1 | protein_coding | blue |
| ADM | protein_coding | blue |
| FCHO1 | protein_coding | blue |
| SLC22A20 | pseudogene | blue |
| SHROOM2 | protein_coding | blue |
| PROM2 | protein_coding | blue |
| PALMD | protein_coding | blue |
| QPRT | protein_coding | blue |
| ATG9B | protein_coding | blue |
| GYG2 | protein_coding | blue |
| WNT4 | protein_coding | blue |
| CBR3 | protein_coding | blue |
| C22orf23 | protein_coding | blue |
| ULBP2 | protein_coding | blue |
| IGSF3 | protein_coding | blue |
| GRHL1 | protein_coding | blue |
| LINC00654 | long_non_coding | blue |
| FAM110C | protein_coding | blue |
| CDH3 | protein_coding | blue |
| CERS4 | protein_coding | blue |
| AL031587.5 | TEC | blue |
| CRYBG2 | protein_coding | blue |
| CLDN1 | protein_coding | blue |
| C5orf38 | protein_coding | blue |
| MT1X | protein_coding | blue |
| MICALL1 | protein_coding | blue |
| ZNF239 | protein_coding | blue |
| IFFO2 | protein_coding | blue |
| KRT80 | protein_coding | blue |
| TTC9 | protein_coding | blue |
| PITX1 | protein_coding | blue |
| HOMER2 | protein_coding | blue |
| EPHA4 | protein_coding | blue |
| GRIP1 | protein_coding | blue |
| IRF6 | protein_coding | blue |
| XKRX | protein_coding | blue |
| EPHB3 | protein_coding | blue |
| PHLDA3 | protein_coding | blue |
| SAMD9 | protein_coding | blue |
| AL031058.1 | long_non_coding | blue |
| MAST4 | protein_coding | blue |
| CSTB | protein_coding | blue |
| KCTD1 | protein_coding | blue |
| TBC1D2 | protein_coding | blue |
| EVPL | protein_coding | blue |
| VLDLR | protein_coding | blue |
| EFNA3 | protein_coding | blue |
| FAM83B | protein_coding | blue |
| HR | protein_coding | blue |
| PITPNM3 | protein_coding | blue |
| DENND5B | protein_coding | blue |
| TACSTD2 | protein_coding | blue |
| SDC1 | protein_coding | blue |
| CEACAM19 | protein_coding | blue |
| GJB3 | protein_coding | blue |
| SLC4A8 | protein_coding | blue |
| TRPS1 | protein_coding | blue |
| ARHGAP23 | protein_coding | blue |
| IFI16 | protein_coding | blue |
| BDKRB1 | protein_coding | blue |
| RHOD | protein_coding | blue |
| TRPV3 | protein_coding | blue |
| DUSP5 | protein_coding | blue |
| DUSP7 | protein_coding | blue |
| ECM1 | protein_coding | blue |
| GSTA4 | protein_coding | blue |
| C3orf67 | protein_coding | blue |
| SRPX2 | protein_coding | blue |
| TSPAN10 | protein_coding | blue |
| SOX7 | protein_coding | blue |
| SLC4A11 | protein_coding | blue |
| SEMA4A | protein_coding | blue |
| MYCL | protein_coding | blue |
| PC | protein_coding | blue |
| BAIAP2 | protein_coding | blue |
| S100A3 | protein_coding | blue |
| ADORA2B | protein_coding | blue |
| PAK6 | protein_coding | blue |
| AL627309.8 | pseudogene | blue |
| SGK1 | protein_coding | blue |
| CCDC170 | protein_coding | blue |
| DLK2 | protein_coding | blue |
| WNT5A | protein_coding | blue |
| TREX2 | protein_coding | blue |
| B3GNT8 | protein_coding | blue |
| AL627309.7 | pseudogene | blue |
| HSPA4L | protein_coding | blue |
| NAV1 | protein_coding | blue |
| DAPP1 | protein_coding | blue |
| MIR210HG | long_non_coding | blue |
| MCIDAS | protein_coding | blue |
| SYTL1 | protein_coding | blue |
| EMP1 | protein_coding | blue |
| AP001453.2 | long_non_coding | blue |
| HCN2 | protein_coding | blue |
| PCAT6 | long_non_coding | blue |
| RNFT2 | protein_coding | blue |
| PRRG4 | protein_coding | blue |
| IRX5 | protein_coding | blue |
| GM2A | protein_coding | blue |
| ALOX12 | protein_coding | blue |
| LYPD5 | protein_coding | blue |
| TNFAIP8L3 | protein_coding | blue |
| MPP7 | protein_coding | blue |
| IKZF2 | protein_coding | blue |
| PGM2L1 | protein_coding | blue |
| ITPRIP | protein_coding | blue |
| KRT10 | protein_coding | blue |
| MPZL2 | protein_coding | blue |
| GDPD1 | protein_coding | blue |
| PERM1 | protein_coding | blue |
| DGKA | protein_coding | blue |
| TYMP | protein_coding | blue |
| SCNN1D | protein_coding | blue |
| PLEKHN1 | protein_coding | blue |
| RARG | protein_coding | blue |
| SPTBN2 | protein_coding | blue |
| TCAF2 | protein_coding | blue |
| ARNTL2 | protein_coding | blue |
| AIF1L | protein_coding | blue |
| SLC2A9 | protein_coding | blue |
| CADM4 | protein_coding | blue |
| EDDM13 | protein_coding | blue |
| S100A16 | protein_coding | blue |
| THSD1 | protein_coding | blue |
| ZSCAN31 | protein_coding | blue |
| SLC6A8 | protein_coding | blue |
| BCL11B | protein_coding | blue |
| DUSP1 | protein_coding | blue |
| IRX2 | protein_coding | blue |
| SLC39A14 | protein_coding | blue |
| MBOAT2 | protein_coding | blue |
| TUBB2A | protein_coding | blue |
| ZNF620 | protein_coding | blue |
| STX11 | protein_coding | blue |
| IMPA2 | protein_coding | blue |
| TSPAN5 | protein_coding | blue |
| FAM43A | protein_coding | blue |
| ACACB | protein_coding | blue |
| AL049555.1 | long_non_coding | blue |
| SLC16A4 | protein_coding | blue |
| TMEM220 | protein_coding | blue |
| CD9 | protein_coding | blue |
| SRD5A1 | protein_coding | blue |
| EHD3 | protein_coding | blue |
| ATP10D | protein_coding | blue |
| CYB5R2 | protein_coding | blue |
| CD44-AS1 | long_non_coding | blue |
| RNF144B | protein_coding | blue |
| SLC6A9 | protein_coding | blue |
| AC004816.1 | long_non_coding | blue |
| ZBED3 | protein_coding | blue |
| B3GNT5 | protein_coding | blue |
| SLC13A4 | protein_coding | blue |
| CRYBG1 | protein_coding | blue |
| CASTOR1 | protein_coding | blue |
| IPPK | protein_coding | blue |
| CCNO | protein_coding | blue |
| GPR153 | protein_coding | blue |
| TBX6 | protein_coding | blue |
| ATP1B3 | protein_coding | blue |
| UPP1 | protein_coding | blue |
| CD302 | protein_coding | blue |
| GAS2L1 | protein_coding | blue |
| RASSF5 | protein_coding | blue |
| TRIM16 | protein_coding | blue |
| MAPKBP1 | protein_coding | blue |
| UNC5B | protein_coding | blue |
| CD44 | protein_coding | blue |
| FAM83F | protein_coding | blue |
| HSD17B1 | protein_coding | blue |
| RNF152 | protein_coding | blue |
| PRRT2 | protein_coding | blue |
| SIPA1L2 | protein_coding | blue |
| FAM213A | protein_coding | blue |
| NUAK2 | protein_coding | blue |
| LRRC8A | protein_coding | blue |
| BAG3 | protein_coding | blue |
| SH3BP1 | protein_coding | blue |
| KAZN | protein_coding | blue |
| MDFI | protein_coding | blue |
| AP001542.3 | long_non_coding | blue |
| JAG1 | protein_coding | blue |
| TNS3 | protein_coding | blue |
| SQLE | protein_coding | blue |
| SEMA3F | protein_coding | blue |
| FUT8 | protein_coding | blue |
| TGFA | protein_coding | blue |
| C1orf74 | protein_coding | blue |
| AC007842.1 | pseudogene | blue |
| TLDC1 | protein_coding | blue |
| OGFRL1 | protein_coding | blue |
| GFOD1 | protein_coding | blue |
| PPP1R13L | protein_coding | blue |
| EXOC6B | protein_coding | blue |
| SOWAHC | protein_coding | blue |
| EPB41L2 | protein_coding | blue |
| FAM83G | protein_coding | blue |
| PIM1 | protein_coding | blue |
| TMEM97 | protein_coding | blue |
| SLC22A23 | protein_coding | blue |
| P3H4 | protein_coding | blue |
| TSKU | protein_coding | blue |
| TLE4 | protein_coding | blue |
| CRNDE | long_non_coding | blue |
| NRGN | protein_coding | blue |
| ITGB8 | protein_coding | blue |
| KLHL23 | protein_coding | blue |
| SLCO3A1 | protein_coding | blue |
| N4BP3 | protein_coding | blue |
| PACSIN3 | protein_coding | blue |
| GPR157 | protein_coding | blue |
| PLS3 | protein_coding | blue |
| ARHGEF37 | protein_coding | blue |
| TUBA4A | protein_coding | blue |
| KLHDC8B | protein_coding | blue |
| IER5 | protein_coding | blue |
| TNFSF10 | protein_coding | blue |
| TTC22 | protein_coding | blue |
| FRRS1 | protein_coding | blue |
| GALC | protein_coding | blue |
| FAM160A1 | protein_coding | blue |
| AP002360.1 | protein_coding | blue |
| SLC51A | protein_coding | blue |
| AMOTL1 | protein_coding | blue |
| TMEM91 | protein_coding | blue |
| CTNNBIP1 | protein_coding | blue |
| EFNB1 | protein_coding | blue |
| ANKRD10-IT1 | long_non_coding | blue |
| GSTP1 | protein_coding | blue |
| SPATA6 | protein_coding | blue |
| AC092611.2 | long_non_coding | blue |
| AC092611.3 | TEC | blue |
| FAM89A | protein_coding | blue |
| PKP3 | protein_coding | blue |
| GKAP1 | protein_coding | blue |
| DGUOK-AS1 | long_non_coding | blue |
| GOLGA2P5 | pseudogene | blue |
| PABPC1L | protein_coding | blue |
| LPAR6 | protein_coding | blue |
| AC008555.8 | TEC | blue |
| CDKN1A | protein_coding | blue |
| PDLIM2 | protein_coding | blue |
| HK1 | protein_coding | blue |
| SCCPDH | protein_coding | blue |
| ZNF233 | protein_coding | blue |
| ALDH4A1 | protein_coding | blue |
| SERPINB8 | protein_coding | blue |
| GLUL | protein_coding | blue |
| TNFAIP8 | protein_coding | blue |
| WDFY2 | protein_coding | blue |
| SPACA9 | protein_coding | blue |
| FAM129B | protein_coding | blue |
| ZFP36 | protein_coding | blue |
| IRF5 | protein_coding | blue |
| AHNAK | protein_coding | blue |
| FDXR | protein_coding | blue |
| DNAJB1 | protein_coding | blue |
| SERTAD2 | protein_coding | blue |
| MAP3K6 | protein_coding | blue |
| PLD1 | protein_coding | blue |
| TRAF5 | protein_coding | blue |
| CITED4 | protein_coding | blue |
| LRRC8E | protein_coding | blue |
| AHDC1 | protein_coding | blue |
| AF001548.2 | long_non_coding | blue |
| PLCD1 | protein_coding | blue |
| STARD5 | protein_coding | blue |
| KCTD11 | protein_coding | blue |
| WDR47 | protein_coding | blue |
| RGS12 | protein_coding | blue |
| THOC3 | protein_coding | blue |
| TEF | protein_coding | blue |
| MREG | protein_coding | blue |
| PPP1R14B | protein_coding | blue |
| BPGM | protein_coding | blue |
