# Supplementary material for: Integrated bioinformatics analysis to decipher molecular mechanism of compound Kushen injection for esophageal cancer by combining WGCNA with network pharmacology
Source: Sci Rep. 2020 Jul 29;10:12745. doi: 10.1038/s41598-020-69708-2 (PMC7391752; doi:10.1038/s41598-020-69708-2)
Supplement: Supplementary file 3 — Supplementary file3 [file 41598_2020_69708_MOESM3_ESM.docx]

**Integrated Bioinformatics Analysis to Decipher Molecular Mechanism of Compound Kushen Injection for Esophageal Cancer by Combining WGCNA with Network Pharmacology**

**Wei Zhou^1^, Jiarui Wu^1^*****, Jingyuan Zhang^1^, Xinkui Liu^1^, Siyu Guo^1^, ShanShan Jia^1^, Xiaomeng Zhang^1^, Yingli Zhu^1^, Miaomiao Wang^1^**

1Beijing University of Chinese Medicine, Beijing, 100102, China

* Corresponding email: exogamy@163.com

**Table S1.** CKI Compound

| No. | Compound | No. | Compound |
| --- | --- | --- | --- |
| 1 | 5, 6-dehydrolupanine | 13 | matrine |
| 2 | 5α, 9α-hydroxymatrine | 14 | N-methylcytisine |
| 3 | 7, 11-dehydromatrine | 15 | oxymatrine |
| 4 | 9α-hydroxymatrine | 16 | oxysophocarpine |
| 5 | 9α-hydroxysophocarpine | 17 | oxysophoranol |
| 6 | adenine | 18 | oxysophoridine |
| 7 | baptifoline | 19 | piscidic acid |
| 8 | isomatrine | 20 | sophocarpine |
| 9 | isosophocarpine | 21 | sophoranol |
| 10 | lamprolobine | 22 | sophoridine |
| 11 | liriodendrin | 23 | trifolirhizin |
| 12 | macrozamin |  |  |
